# ST6GalNAc‐I promotes lung cancer metastasis by altering MUC5AC sialylation

**DOI:** 10.1002/1878-0261.12956

**Published:** 2021-05-01

**Authors:** Imayavaramban Lakshmanan, Sanjib Chaudhary, Raghupathy Vengoji, Parthasarathy Seshacharyulu, Satyanarayana Rachagani, Joseph Carmicheal, Rahat Jahan, Pranita Atri, Ramakanth Chirravuri‐Venkata, Rohitesh Gupta, Saravanakumar Marimuthu, Naveenkumar Perumal, Sanchita Rauth, Sukhwinder Kaur, Kavita Mallya, Lynette M. Smith, Subodh M. Lele, Moorthy P. Ponnusamy, Mohd W. Nasser, Ravi Salgia, Surinder K. Batra, Apar Kishor Ganti

**Affiliations:** ^1^ Department of Biochemistry and Molecular Biology University of Nebraska Medical Center Omaha NE USA; ^2^ Department of Biostatistics College of Public Health University of Nebraska Medical Center Omaha NE USA; ^3^ Department of Pathology and Microbiology University of Nebraska Medical Center Omaha NE USA; ^4^ Eppley Institute for Research in Cancer and Allied Diseases Omaha NE USA; ^5^ Fred & Pamela Buffett Cancer Center University of Nebraska Medical Center Omaha NE USA; ^6^ Department of Medical Oncology and Therapeutics Research City of Hope Comprehensive Cancer Center Beckman Research Institute Duarte CA USA; ^7^ Department of Internal Medicine VA Nebraska Western Iowa Health Care System University of Nebraska Medical Center Omaha NE USA

**Keywords:** FAK, integrin β4, lung cancer metastasis, MUC5AC, ST6GalNAc‐I

## Abstract

Lung cancer (LC) is the leading cause of cancer‐related mortality. However, the molecular mechanisms associated with the development of metastasis are poorly understood. Understanding the biology of LC metastasis is critical to unveil the molecular mechanisms for designing targeted therapies. We developed two genetically engineered LC mouse models Kras^G12D/+^; Trp53^R172H/+^; Ad‐Cre (KPA) and Kras^G12D/+^; Ad‐Cre (KA). Survival analysis showed significantly (*P* = 0.0049) shorter survival in KPA tumor‐bearing mice as compared to KA, suggesting the aggressiveness of the model. Our transcriptomic data showed high expression of N‐acetylgalactosaminide alpha‐2, 6‐sialyltransferase 1 (St6galnac‐I) in KPA compared to KA tumors. ST6GalNAc‐I is an O‐glycosyltransferase, which catalyzes the addition of sialic acid to the initiating GalNAc residues forming sialyl Tn (STn) on glycoproteins, such as mucins. Ectopic expression of species‐specific p53 mutants in the syngeneic mouse and human LC cells led to increased cell migration and high expression of ST6GalNAc‐I, STn, and MUC5AC. Immunoprecipitation of MUC5AC in the ectopically expressing p53^R175H^ cells exhibited higher affinity toward STn. In addition, ST6GalNAc‐I knockout (KO) cells also showed decreased migration, possibly due to reduced glycosylation of MUC5AC as observed by low STn on the glycoprotein. Interestingly, ST6GalNAc‐I KO cells injected mice developed less liver metastasis (*P* = 0.01) compared to controls, while colocalization of MUC5AC and STn was observed in the liver metastatic tissues of control mice. Collectively, our findings support the hypothesis that mutant p53^R175H^ mediates ST6GalNAc‐I expression, leading to the sialyation of MUC5AC, and thus contribute to LC liver metastasis.

## Graphical abstract

In the presence of mutant p53^R175H^, ST6GalNAc‐I mediates MUC5AC sialylation that increases the aggressiveness of lung adenocarcinoma (LUAD). MUC5AC sialylation is required for integrin β4 interaction that induces angiogenesis and liver metastasis. Hence, targeting the ST6GalNAc‐I/MUC5AC could prevent LUAD metastasis.

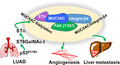

**Abbreviations**
Ad‐Cre: AdenoCre
BAG: benzyl‐N‐acetyl‐α‐galactosaminide
FAK: focal adhesion kinase
IHC: immunohistochemistry
IP: immunoprecipitation
IVIS: *in vivo* live imaging
KA: Kras^G12D^, Ad‐Cre
KD: knockdown
KO: knockout
KPA: Kras^G12D/+^, Trp53^R172H/+^, Ad‐Cre
LC: lung cancer
LUAD: lung adenocarcinoma
MUC: mucins
NSCLC: non‐small‐cell lung cancer
PCR: polymerase chain reaction
ST6GalNAc‐I: N‐acetylgalactosaminide alpha‐2, 6‐sialyltransferase I
STn: sialyl Tn
TCGA: The Cancer Genome Atlas
TMA: tissue microarray
TP53: tumor protein 53
Trp53: transformation‐related protein 53
UNMC: University of Nebraska Medical Center

## 1 Introduction

Lung cancer (LC) is the most common cause of cancer‐related death worldwide. In the United States alone, 235 760 new LC cases are expected, which would constitute about 22.5% of all cancer‐related deaths in 2021 [1]. Despite recent advances, the overall 5‐year survival rate remains dismal for LC patients [1]. Approximately 30–40% of non‐small‐cell lung carcinoma (NSCLC) patients develop liver metastases with a median survival of only 8 months [2]. *p53* tumor suppressor gene is frequently lost or mutated in cancer, associated with invasive and metastatic potential [3, 4, 5]. In LC, p53 mutations may contribute to the molecular mechanism of metastasis and could serve as a therapeutic target [6, 7]

ST6GalNAc‐I (N‐acetylgalactosaminide alpha‐2, 6‐sialyltransferase 1) is a mucin‐type O‐glycosyltransferase, which catalyzes the addition of sialic acid to first sugar GalNAc (Tn) and results in the formation of Neu5Acα2,6GalNAc (sialyl Tn; STn) carbohydrate antigen [8, 9]. Functional studies have demonstrated ST6GalNAc‐I to play a critical role in cancer cell growth and migration by altering the O‐glycosylation pattern of glycoproteins [10, 11, 12]. Several studies have shown cancer‐associated STn to strongly associate with disease aggressiveness and poor prognosis [9, 13, 14, 15]. Recent studies have also shown that ST6GalNAc‐I is overexpressed in NSCLC and functions as a biomarker to distinguish lung adenocarcinoma (LUAD) from lung squamous cell carcinoma, suggesting the critical role of ST6GalNAc‐I in LUAD pathobiology [16].

Mucins (MUC) are the preferential substrates for O‐glycosylation, owing to their central domain rich in serine and threonine residues (sites for O‐glycosylation) [17, 18]. The profile of glycan moieties dictates the functions of glycoproteins; and aberrant glycosylation leads to tumor progression and metastasis [19, 20]. Increased expression of ST6GalNAc‐I leads to augmented STn expression, which has been associated with enhanced metastasis through mucin signaling [21, 22]. Previously, we have reported that MUC5AC and MUC16 MUC are involved in the growth and metastasis of LC cells [23, 24]. Here, we test the hypothesis that differential expression of ST6GalNAc‐I observed in p53 mutated LUAD alters MUC5AC glycosylation, resulting in liver metastasis.

## 2 Materials and methods

### 2.1 Development of a spontaneous lung cancer model and RNA sequence analysis

Genetically engineered mouse (GEM) models for LC were generated by crossing LSL‐Kras^G12D/+^ (B6.129‐Krastm4Tyj (01XJ6)) with Trp53^R172H/+^. The primers used for genotyping (*Kras* and *Trp53*) were mentioned in Table S1. Kras^G12D/+^; Trp53^R172H/+,^ and Kras^G12D^ mice were infected with an Ad‐Cre‐Luciferase retroviral vector intranasally (University of Iowa, Gene and Vector Core, IA, USA) or with vector control. Four weeks post‐infection, the animals were injected with luciferin intra‐peritoneally to monitor the tumor growth by noninvasive *in vivo* live imaging (IVIS) imaging system [24, 25]. Mice were supplied with food and water *ad libitum* and subjected to a 12‐h light/dark cycle. The mouse studies were performed in accordance with the US Public Health Service ‘Guidelines for the Care and Use of Laboratory Animals’ under an approved protocol by the Institutional Animal Care and Use Committee, University of Nebraska Medical Center.

### 2.2 Cell culture and transfection

A549, H292, and H1437 LC cells purchased from ATCC were cultured in RPMI medium supplemented with 10% FBS and antibiotics [25]. Similarly, mouse tumor cell line K1418 (established from our GEM models) [24] was also cultured in DMEM medium with the above‐mentioned supplements. The cells were incubated in a humidified atmosphere at 37 °C with 5% CO_2_. HUVEC cells were cultured and maintained as described in ATCC (https://www.atcc.org/products/all/CRL‐1730.aspx). Lox‐stop‐Lox p53^R172H^ (Addgene, Watertown, MA, USA; Plasmid #14854) and pLenti6/V5‐p53_R175H (TP53^R175H^) (Addgene, plasmid #22936) were used for mutant p53 transfection experiments. ST6GalNAc‐I KO was performed using the CRISPR Cas9 method (ST6GalNAc1 CRISPR guide RNA 2 cloned in pSpCas9 BB‐2A‐GFP (PX458) vector). The guide RNA sequence (GGCCAACCAGGCACCGCCGG) was used for targeting ST6GalNAc‐I. Endogenously expressing MUC5AC was knocked down using a small hairpin RNA construct (pSUPER‐Retro‐shMUC5AC) by a stable transfection method [25].

### 2.3 Tissue Microarray and immunohistochemistry

We used commercially available tissue microarray (TMA) (Cat#HLugA150CS02; US Biomax, Rockville, MD, USA), which included 75 cases of LUAD and normal lung tissues. The TMA was analyzed for ST6GalNAc‐I (Cat#ab82821), MUC5AC [45M1] (Cat#ab3649; Abcam, Cambridge, MA, USA), and STn (Cat#LS‑C170901; clone B35.1; LSBio, Seattle, WA, USA) expression by immunohistochemistry (IHC), as described previously [25].

### 2.4 Immunoblot and sandwich ELISA analysis

Western blot assay was performed in the whole‐cell lysate (WCL) isolated in radioimmunoprecipitation assay buffer (50 mm Tris‐/HCl, 150 mm NaCl, 1% NP‐40, 0.5% sodium deoxycholate along with protease inhibitor cocktail) as described previously [23]. About 20–40 µg of WCL was resolved in 10–12% SDS/PAGE gel, and MUC were resolved in 2% SDS agarose gel. Blots were transferred in PVDF membrane, blocked in 5% skimmed milk, washed with Tris‐buffered saline‐Tween‐20 (TBS‐T, 3×, 10 min), and incubated with the following primary antibodies: ST6GalNAc‐I (Cat#ab82821), MUC5AC (CLH2 Cat#MAB2011; Millipore, Burlington, MA, USA), pFAK (Y397) (Cat#3283), integrin α6, β1, β3, β4, β5 (Cat#4749; Cell Signaling Technology, Danvers, MA, USA), and β‐actin (Cat#A1978; Sigma, St Louis, MO, USA). The membranes were then washed (3×, 10 min) in TBS‐T, incubated with the respective secondary antibodies for 1 h at room temperature, and subsequently washed with PBST (3×, 10 min). The signals were detected with the ECL chemiluminescence kit (GE Healthcare Bio‐Sciences, Pittsburgh, PA, USA). Secretory levels of MUC5AC in the culture supernatant were quantified by sandwich ELISA as described previously [26].

### 2.5 Immunoprecipitation analysis

STn, MUC5AC (CLH2), MUC4 (8G7), and MUC16 (M11 clone) antibodies were incubated overnight with WCLs (500 μg) isolated from A549 cells transfected with p53^R175H^ or control, in a 750 μL total volume. Protein A + G Sepharose beads were added to the lysate‐antibody mix and incubated on a rotating platform for 4 h at 4 °C and then washed four times with immunoprecipitation (IP) assay buffer [25]. The immunoprecipitants and input were electrophoretically resolved on 2% SDS agarose. The membranes were blocked in 5% skimmed milk in TBS‐T for at least 1 h and then incubated with respective antibodies. The signals were detected with the ECL chemiluminescence kit.

### 2.6 Tube formation assay

Human umbilical vein endothelial cells (HUVECs; 2.0 × 10^4^) were plated on Matrigel‐coated 96‐well plates (100 μL/well) cultured with the conditioned media (collected from A549 cells), and tube formation was evaluated as described earlier [27]. Images were analyzed using angio tool 64 0.6a software (https://sites.imagej.net/AngioTool/) avaiable in ImageJ (https://imagej.net).

### 2.7 Scoring and statistical analysis

ST6GalNAc‐I, MUC5AC, and STn immunostaining intensity were evaluated by a trained pathologist (SML) who was blinded to the clinical information. Each sample was given a composite score based on the percentage of positive cells and intensity and extent of tissue staining using specific antibodies. Intensity was graded on a four‐point scale: − (0), + (1), ++ (2) and +++ (3). Extent of staining was graded as: 1 (0–24%), 2 (25–49%), 3 (50–74%), and 4 (75–100%). A composite score was obtained by multiplying the two values. Quantitative assessment of ST6GalNAc‐I and MUC5AC protein expression in the xenograft tissues was performed using fijiimage j software (https://imagej.net/Fiji). DAB‐stained A549 scramble control and ST6GalNAc‐I knockout (KO) xenograft tissues were photographed using Leica light microscope (Buffalo Grove, IL, USA), and semi‐quantitative IHC images were automatically scored using the plugins associated with fijiimagej software as described previously [28].

### 2.8 Quantitative real‐time PCR

Quantitative real‐time PCR (QRT‐PCR) performed as previously described [24]. Total RNA was isolated using Qiagen Kit (Germantown, MD, USA). Total RNA (2 µg) of total RNA was used for cDNA synthesis using reverse transcriptase SuperScript®II (Invitrogen, Carlsbad, CA, USA). Quantitative PCR was performed using SYBER Green, and β‐actin was used as an internal control. The data were calculated based on the $2^{‐\Delta \Delta C_{t}}$ method. All primers used in the study are described in Table S1.

### 2.9 Cell motility assay

The control and Trp53^R172H^/p53^R175H^‐transfected cell lines (K1418, A549, and H292) and ST6GalNAc‐I KO cells and respective control (A549) were seeded (1 × 10^6^ cells) on top of the Boyden chamber (8 μm) pore size in serum‐free medium. Complete medium with 20% serum used as a chemoattractant was added on the bottom of the insert (six‐well plate) and allowed to migrate for 24 h. The migrated cells were stained with Diff‐Quick stain and counted at different fields of vision.

### 2.10 Immunofluorescence

About 25 000 cells (A549 Control and ST6GalNAc‐I KO) were grown over the sterile coverslips in a six‐well plate for 48 h. Cells were washed with HBSS (3×, 5 min), fixed with 4% paraformaldehyde (10 min, RT), washed with PBS (3×, 5 min), and blocked with 10% normal goat serum for 1 h. STn/MUC5AC or MUC5AC/Integrin β4 antibodies were then added to the respective samples for overnight at 4 °C. The next day, the cells were washed with PBS and incubated with respective secondary antibodies (Alexa Fluor 488; Cat#A‐11034 and Alexa Fluor 568; Cat#A‐11004; Thermo Fisher Scientific, Waltham, MA, USA) for 1 h, RT. Finally, the cells were washed with PBS, and the coverslips were mounted on the slides with an anti‐fade Vectashield mounting medium (Vector Laboratories, Burlingame, CA, USA) containing 4′,6‐diamidino‐2‐phenylindole (DAPI). All images were acquired with LSM710 confocal microscope (Carl Zeiss, Rudolf‐Eber‐Strasse 2, Oberkochen, Germany). For immunofluorescence in tissue sections, the slides were washed with xylene (4×10 min) and serially hydrated with an alcohol solution (100%, 90%, 70%, 50%, 30%, and 20%) for 10 min each. Antigen retrieval was done in 10mM sodium citrate buffer; 0.05% Tween 20 (P^H^ 6.0) in the microwave for 15 min, washed, and blocked with 2.5% horse serum (Impress Reagent Kit, Vector Laboratories, Burlingame, CA, USA). The respective steps were followed as mentioned above.

### 2.11 Proximity ligation assay

Proximity ligation was performed using Duolink™ In Situ Red Starter Kit Mouse/Rabbit (Cat#DUO92101, Sigma) according to the manufacturer’s instructions. Briefly, 0.1 × 10^6 ^cells (A549‐p53^R175H^ transfected or A549‐St6GalNAc‐I KO and respective control cells) were seeded on cover slip in the 12‐well plate for 48 h. The cells were then washed with HBSS (3×, 5 min), fixed with 4% paraformaldehyde (10 min, RT), and washed further with PBS (3×, 5 min). The samples are then blocked with blocking solution (30 min, RT), and respective primary antibodies [MUC5AC (45M1), Cat#ab3649 and integrin β4, Cat#ab133682 (Abcam); Sialyl Tn, Cat#LS‐C170901] diluted in antibody diluent were added. After overnight incubation, the cells were washed with wash buffer A (2×, 5 min); diluted probes (1 : 5) were added for 1 h, RT. The probes were then ligated with ligase (30 min/ RT) and amplified. Finally, the samples were washed with wash buffer B (2×, 10 min), air‐dried, and mounted with ProLong Gold anti‐fade reagent containing DAPI. Images were acquired by confocal microscope (LSM710).

### 2.12 Data analysis

Statistical significance was evaluated with the Student's *t*‐test and ordinary one‐way ANOVA followed by Tukey’s multiple comparisons test using graphpad prism software (GraphPad Software, version 8.1.2, San Diego, CA, USA). Differences between groups were considered to be statistically significant when the *P*‐value was < 0.05. All experiments were performed in triplicates. Data represents mean ± SD.

## 3 Results

### 3.1 Presence of mutant Trp53 in lung adenocarcinoma correlates with poor survival

**Fig. 1:**
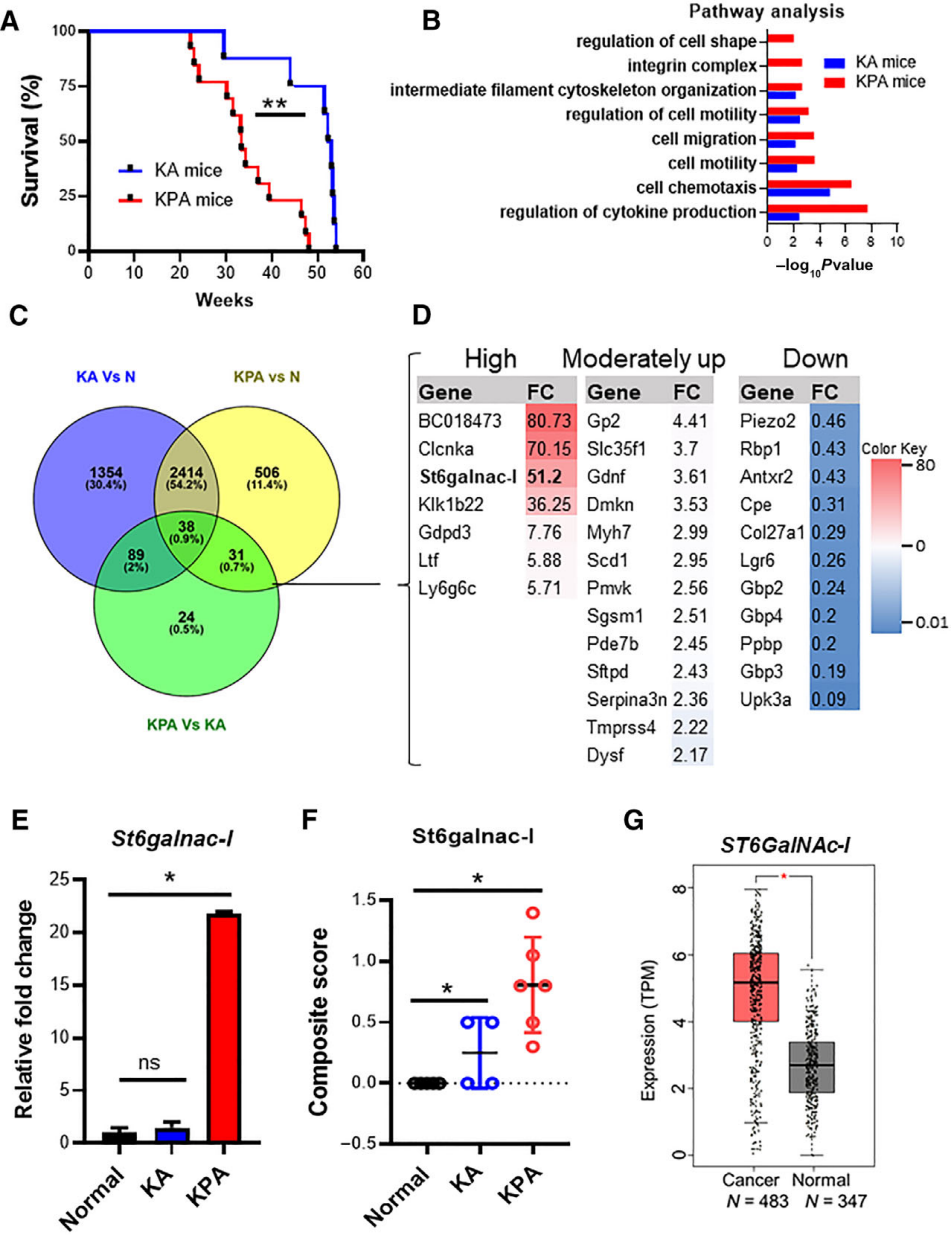
Development of a lung cancer mouse model harboring Kras^G12D/+^; Trp53^R172H/+^ by Ad‐Cre. (A) We have developed two different Ad‐Cre‐mediated LC mouse models, one harboring Kras^G12D^ (KA) and the other harboring Kras^G12D^; Trp53^R172H^ (KPA). Kaplan–Meier survival curves displaying the overall survival of KPA (*n* = 13) and KA (*n* = 8) tumor‐bearing mice (blue line KA and red line KPA). (B) Pathway enrichment in KPA and KA tumors based on differential gene expression analyzed using the ConsensusPathDB tool. (C) Venn diagram representing genes that are expressed in KA and KPA tumors with respect to normal lung tissues was generated by Venny 2.1. Unique genes (31) specific only to KPA were identified. These genes were further categorized based on the extent of gene expression (logFC) as (D) highly, moderately up, and downregulated genes. (E) Quantitative PCR analysis showing increased expression of *St6galnac‐I* in the KPA tumors (*n* = 4) compared with normal lung (*n* = 4) and KA tumors (*n* = 4). (F) Dot plot demonstrating composite score of immunopositivity for ST6GalNAc‐I protein in the normal lung (*n* = 5), KA (*n* = 4), and KPA (*n* = 6) tumors by IHC. (G) Box plot depicting increased expression of *ST6GalNAc‐I* (transcript) in the normal (*n* = 483) and LC (*n* = 347) tissues in TCGA‐LUAD dataset. TPM, transcripts per million. β‐Actin was used as an internal control. Statistical significance * *P* < 0.05, ** *P* < 0.01. All experiments were performed in triplicates. Error bars represent the mean ± SD. Statistical significance was tested using two‐tailed *t*‐test (A, E, F, & G).

We used GEM models of LC by activation of Kras^G12D^ and Trp53^R172H^ mutations via Ad‐Cre inhalation, as described previously [24, 25]. Comparing Kras^G12D/+^; Trp53^R172H/+^; Ad‐Cre (KPA) and Kras^G12D/+^; Ad‐Cre (KA) mice, we observed a significantly shorter survival in KPA tumor‐bearing mice (*P* = 0.0049) as compared to KA mice (Fig. 1A). These data indicate that KPA driven tumors are more aggressive than the KA tumors. Correspondingly, the KPA LUAD tissues exhibited a relatively high expression of proliferation marker Ki67 (Fig. S1A) compared with KA lung tumors. Furthermore, KA and KPA tumor tissues expressed high LUAD marker keratin 7 (Fig. S1B), whereas keratin 5 (squamous‐type marker) expression was low (Fig. S1C), confirming that Kras^G12D/+^; Trp53^R172H/+^ mutants develop LUAD.

### 3.2 Mutant Trp53^R172H^‐specific gene signatures and signaling pathways in LUAD

To identify the *Trp53* ^R172H^‐specific molecular signatures responsible for disease aggressiveness, RNA‐Seq studies were performed in the KPA (*n* = 3), KA tumor (*n* = 3), and normal lung tissues (*n* = 3). The KA and KPA genes were segregated, followed by normalization with littermate control lung tissues. We performed the functional enrichment analysis using the identified unique genes with ConsensusPathDB tool (https://cpdb.molgen.mpg.de/). We identified that genes related to cell migration, motility, cytoskeleton organization, chemotaxis, integrins, and cytokine production pathways were significantly activated in the KPA tumors compared to KA (Fig. 1B) suggesting its role in the aggressive behavior of these tumors.

### 3.3 Trp53^R172H^ mutation causes the overexpression of St6galnac‐I in LUAD

Based on the Venn diagram analysis using Venni software (https://bioinfogp.cnb.csic.es/tools/venny/), we observed KA (1354) and KPA (506)‐specific genes (Fig. 1C), while 2414 genes were common between the KA and KPA tumors. We observed 31 genes uniquely expressed in the KPA tumors, indicating that mutant Trp53^R172H^ may possibly regulate these 31 genes in LUAD. We segregated these 31 genes based on: high (seven genes), moderately upregulated (13 genes), and downregulated (11 genes) (Fig. 1D). We validated the clinical relevance of the seven highly upregulated genes in LUAD (LUNG CANCER EXPLORER). We found that *ST6GalNAc‐I* (*P* = 4.2e‐14) (Fig. S1D) to be among the top differentially expressed genes in LUAD patients compared to healthy individuals (https://lce.biohpc.swmed.edu/lungcancer/). *St6galnac‐I* transcripts were significantly higher in the KPA tumors (*P* = 0.02) than KA and normal lung tissues (Fig. 1E). We further validated the St6galnac‐I expression by IHC in the KPA (*n* = 5), KA (*n* = 4), and normal (*n* = 5) murine lung tissues and found a significantly high reactivity in KPA (*P* = 0.03) compared to compared to KA and normal (Fig. 1F). Furthermore, in silico The Cancer Genome Atlas (TCGA) analysis confirmed the significantly high expression of *ST6GalNAc‐I* in LUAD samples (*n* = 483) compared to healthy individuals (*n* = 347) (Fig. 1G) (https://gepia.cancer‐pku.cn/). Furthermore, we also observed increased median expression of *ST6GalNAc‐I* in the p53^R175H^ mutated LC patient samples compared to tumor protein 53 (TP53) wild‐type counterparts (Fig. S1E). These findings suggest that Trp53^R172H^ mutation potentially enhances the aggressive nature of LUAD by elevating the St6galnac‐I levels.

### 3.4 Expression of mutant Trp53^R172H^ in syngeneic KA cells and its impact on St6galnac‐I expression and cell motility

**Fig. 2:**
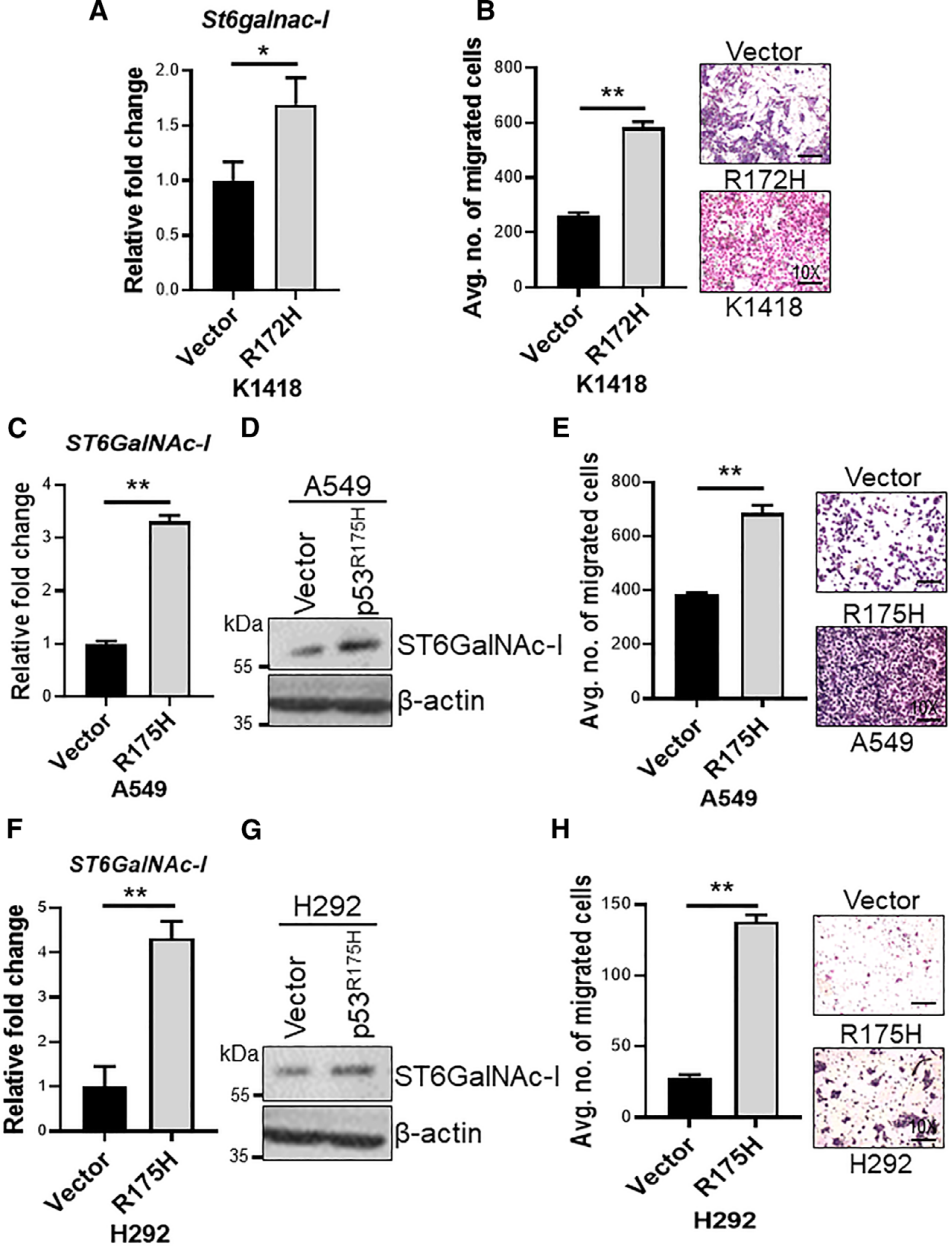
Overexpression of mutant p53 upregulates ST6GalNAc‐I in lung cancer cells. (A) Quantitative PCR depicting increased *St6galnac‐I* expression after mutant Trp53^R172H^ transfection in K1418 (derived from KA tumor) mouse LC cells compared with respective vector‐transfected control (*n* = 3). (B) Bar graph showing increased migration of mouse LC cells (K1418) transfected with mutant Trp53^R172H^ compared with vector control. Representative images of Boyden chamber transwell migration assay showing increased migratory of potential of mouse LC cells in the mutant Trp53^R172H^ transfected cells compared to control (*n* = 3). (C) Quantitative PCR (bar diagram) (*n* = 3) and (D) western blot analysis showing increased expression of ST6GalNAc‐I upon ectopic expression of mutant p53 (R175H) in A549 cell line. (E) Bar diagram indicating increased migratory potential of A549 cells transfected with mutant p53 (R175H) compared with vector control (*n* = 3). Representative images (10×) of Boyden transwell migration assay are provided (right panel). (F) Similarly, quantitative PCR (bar diagram) (*n* = 3) and (G) western blot showing increased expression of ST6GalNAc‐I upon ectopic expression of mutant p53 (R175H) in H292 cell line. (H) Bar diagram showing increased cell migration of H292 cells transfected with mutant p53 (R175H) cells compared to vector controls (*n* = 3). Respective representative images (10X) of Boyden chamber transwell migration assay (right panel) are provided. β‐actin used as a loading control. Statistical significance * *P* < 0.05; ** *P* < 0.01. All experiments were performed in triplicates. Error bars represent the mean ± SD. Statistical significance was tested using two‐tailed *t*‐test (A, B, C, E, F, & H).

To determine the impact of mutant Trp53^R172H^ on *St6galnac‐I* expression in LUAD, we ectopically expressed the mutant Trp53^R172H^ in the KA tumor syngeneic cell line (KA1418 has wild‐type Trp53 derived from Kras^G12D^‐activated tumor). We observed that Trp53^R172H^ mutation significantly increased the *St6galnac‐I* expression compared to control cells (Fig. 2A). Simultaneously, the mutant Trp53^R172H^ transfected KA1418 cell line showed significantly high migratory potential (*P* = 0.008) compared to controls (Fig. 2B).

### 3.5 Mutant p53^R175H^ expression in human lung cancer cells and its impact on ST6GalNAc‐I expression and function

To evaluate the impact of p53^R175H^ on ST6GalNAc‐I expression, we ectopically expressed p53^R175H^ in the LC cell lines A549 (Kras mutated and p53 wild‐type) and H292 (Kras/p53 wild‐type) and observed that p53^R175H^ activation leads to increased expression of ST6GalNAc‐I (both mRNA and protein) in A549 (Fig. 2C,D) and H292 cells (Fig. 2F,G) compared to controls. Additionally, p53^R175H‐^transfected A549 (*P* = 0.04*)* and H292 (*P* = 0.009*)* cells also demonstrated significantly high motility compared to control cells (Fig. 2E,H), suggesting that mutant p53^R175H^ is a causative player for ST6GalNAc‐I mediated LC cell migration.

### 3.6 Mutant p53^R175H^ induced MUC5AC glycosylation through ST6GalNAc‐I

**Fig. 3:**
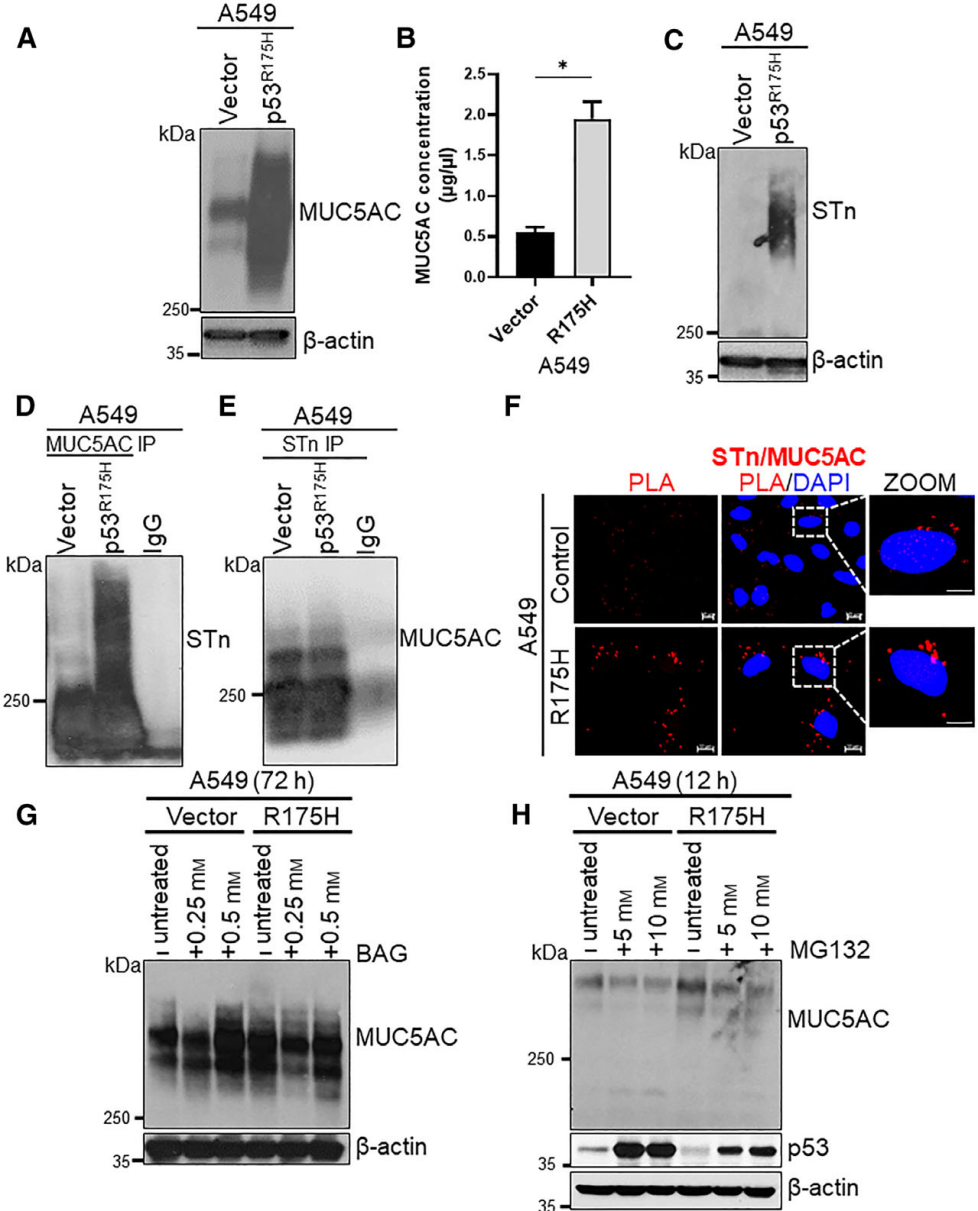
Mutant p53^R175H^ mediates MUC5AC glycosylation via ST6GalNAc‐I. (A) 2% SDS agarose gel (40 µg total lysates) showing increased MUC5AC (CLH2 antibody) in the p53 mutant (R175H) transfected A549 cells compared with controls. (B) Bar diagram showing increased secretory MUC5AC (microgram per microliter) determined by sandwich ELISA in the culture supernatant of A549 cells transfected with p53^R175H^ mutant and vector control (*n* = 3). (C) STn levels in the mutant p53^R175H^‐transfected A549 and control cells by Western blot analysis (2% SDS agarose). (D) MUC5AC immunoprecipitated in the mutant p53^R175H^‐transfected and control cells, and probed with STn antibody. (E) STn immunoprecipitated in the mutant p53^R175H^‐transfected and control cells and probed with MUC5AC. (F) PLA showing increased interaction (red fluorochrome) between STn and MUC5AC in the p53 mutant (R175H) transfected and control A549 cells. (G & H) Western blot showing MUC5AC expression in LC cells treated with BAG (0.25 and 0.5 mm, 72 h) and MG132 (0.5 and 10 mm, 12 h). β‐actin used as a loading control. Statistical significance * *P* < 0.05. Error bar represents mean ± SD of experiments performed in triplicates. Two‐tailed *t*‐test was used for statistical significance (B).

We next analyzed the expression of MUC, including MUC4, MUC5AC, and MUC16 in the p53^R175H^ mutant transfected LC cells. We found increased levels of MUC5AC (Fig. 3A) and MUC16 expression (Fig. S2A) in the mutant p53‐transfected cells than control. However, we observed no significant difference in the MUC4 expression in the mutant p53 transfected compared to control cells (Fig. S2B). As MUC5AC is a secretory glycoprotein, we assessed its secretion levels by sandwich ELISA in the culture supernatant collected from p53^R175H^ mutant transfected and vector control A549 cells. Similar to increased MUC5AC protein expression, there were also significantly high levels of secretory MUC5AC in the culture supernatant of mutant p53‐transfected cells (*P *= 0.01) (Fig. 3B). Importantly, increased levels of STn were observed in the ectopically expressing p53^R175H^ mutant cell line compared to control (Fig. 3C). To further assess whether the glycosylation status of mucin is due to mutant p53^R175H^, we immunoprecipitated various MUCs‐ MUC4 (8G7), MUC5AC (CLH2), and MUC16 (M11 clone) antibodies in the WCLs of p53^R175H^ mutant transfected cells (A549 and H292) and their respective control cells, and then probed with STn‐detecting antibody. We observed an increased level of STn on the MUC5AC in p53 mutant transfected cells where ST6GalNAc‐I expression was also high (Fig. 3D,E). We further corroborated the co‐IP data by proximity ligation assay (PLA), where we found increased levels of STn on MUC5AC in the p53^R175H^ mutant A549 cells compared to control (Fig. 3F). No change in STn levels was observed in the MUC16 and MUC4 immunoprecipitated from mutant p53^R175H^ vs. control cells (Fig. S2C,D). Although several regulators such as *NFkB*, *SP1*, and *Gli1* are known to regulate MUC5AC expression [29, 30, 31], we observed a significant increase in the expression of *NFkB* alone, in the p53 mutant transfected A549 cells compared to vector controls (Fig. S2E‐G). However, there was no significant difference in the transcripts of *MUC5AC* after ectopic expression of mutant p53 (Fig. S2H) and control cells suggesting that NFkB might not be involved in the modulation of MUC5AC in mutant p53^R175H^ driven LC tumorigenesis. These findings suggest that p53^R175H^ mutant induced the ST6GalNAc‐I expression to increase STn moieties on the MUC5AC glycoprotein.

### 3.7 Effect of O‐glycosylation and proteasome inhibitor on MUC5AC expression in mutant p53^R175H^ cells

Benzyl‐N‐acetyl‐α‐galactosaminide (BAG) is a competitive inhibitor of enzymes using N‐acetylgalactosamine as an acceptor [32]. To determine the propensity of MUC5AC to be O‐glycosylated, we treated the p53^R175H^ mutant and vector control LC cells with BAG at different concentrations (0.25 and 0.5 mm) for 72 h. BAG treatment led to a variation in the band pattern of MUC5AC compared to untreated cells, suggesting that differential O‐glycosylation of MUC5AC in LC cells transfected with p53^R175H^ mutant (Fig. 3G). Next, to determine whether the high level of MUC5AC observed upon ectopic p53 mutant expression was due to protein stability, we treated the p53 mutant and vector control cells with MG132, a proteasomal inhibitor (MG132) at various concentrations (5 and 10 mm) for 12 h. No change in the MUC5AC levels at protein level was detected (Fig. 3H). These findings suggest that higher level of MUC5AC in the mutant p53 cells is mainly due to differential glycosylation and not due to increased protein stability.

### 3.8 Expression of ST6GalNAc‐I, STn, and MUC5AC in lung cancer patient samples

**Fig. 4:**
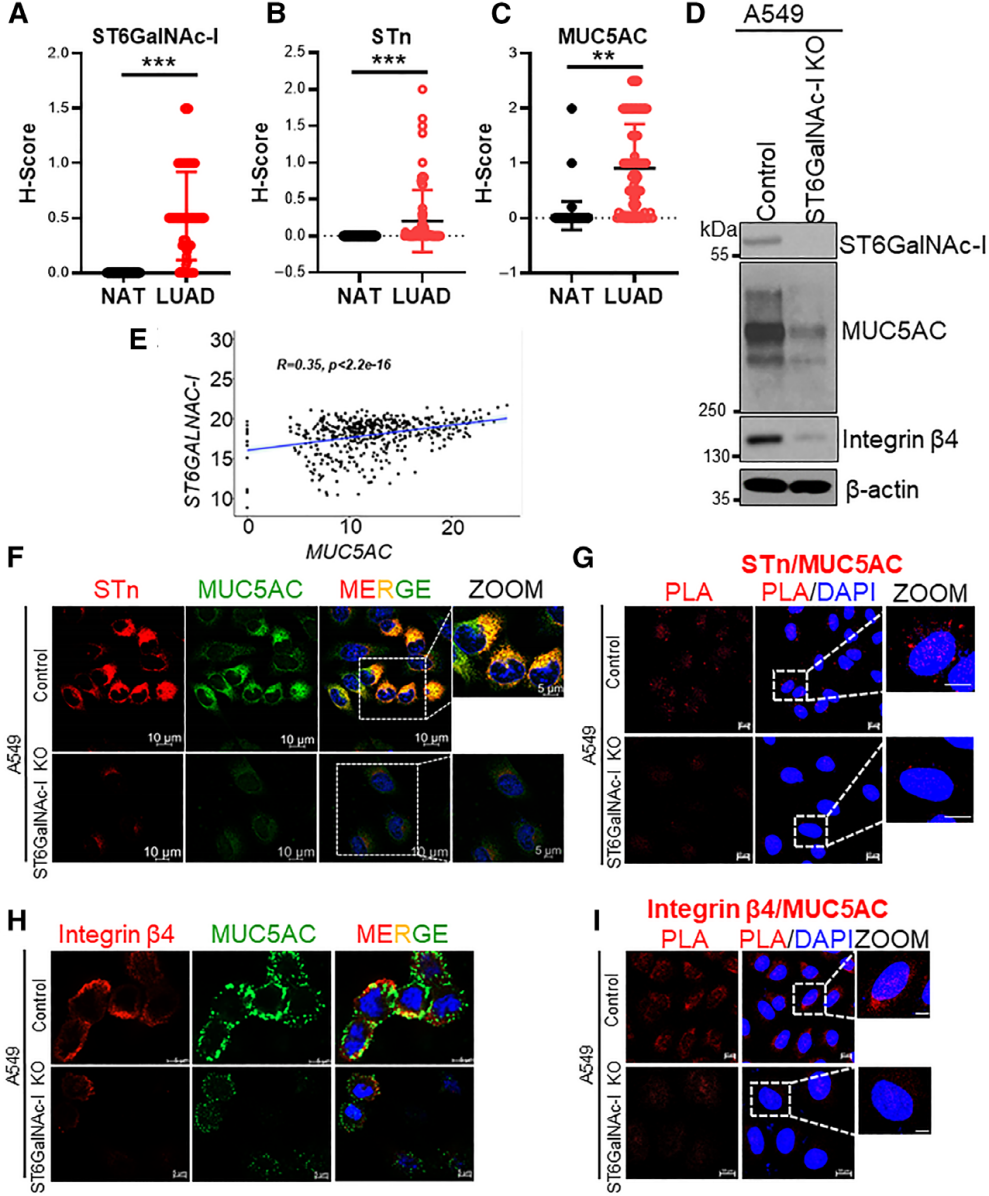
Expression of ST6GalNAc‐I, STn, and MUC5AC in LUAD. (A‐C) Immunohistochemical analysis showing overexpression (*H*‐score) of (A) ST6GalNAc‐I, (B) STn, and (C) MUC5AC in LUAD tissues (*n* = 75) compared to normal lung (*n* = 75). (D) Western blot analysis showing decreased expression of ST6GalNAc‐I, MUC5AC, and integrin β4 in the ST6GalNAc‐I KO cells compared to A549 control. β‐actin used as a loading control. (E) Correlation plot showing positive correlation between ST6GalNAc‐I and MUC5AC expression in TCGA‐LUAD (*n* = 347). (F) Co‐expression of MUC5AC and STn in the control and ST6GalNac‐I KO cells analyzed by immunofluorescence and (G) PLA. (H) Immunofluorescence and (I) PLA showing colocalization of integrin β4 and MUC5AC in A549 cells, while minimal interaction was observed in the ST6GalNAc‐I KO cells. Statistical significance **P < 0.01; *** *P* < 0.001. Error bar represents mean ± SD. Statistical significance was tested using two‐tailed *t*‐test (A‐C).

We performed IHC in the TMAs containing 75 cores of LUAD tissues and 75 cores of normal adjacent tissues (NAT). We stained for ST6GalNAc‐I, STn, and MUC5AC in three separate TMAs and observed significantly high levels of ST6GalNAc‐I, STn, and MUC5AC in LUAD (Fig. 4A‐C) but minimal or undetectable in NAT (Fig. 4A‐C). Furthermore, both ST6GalNAc‐I and MUC5AC were elevated in stage IV compared to early stages of the disease (Fig. S3A,B). We further observed co‐expression of ST6GalNAc‐I and MUC5AC in LUAD patients (Fig. S3C). This finding was further corroborated by positive correlation observed between ST6GalNAc‐I and MUC5AC expression in the LUAD samples of TCGA‐LUAD dataset (Fig. 4E).

### 3.9 Impact of ST6GalNAc‐I on MUC5AC sialylation

To determine the role of ST6GalNAc‐I in mucin glycosylation and its role in cancer cell aggressiveness, we performed CRISPR‐Cas9‐based KO of *ST6GalNAc‐I* in A549 and H1437 cells. KO of ST6GalNAc‐I was confirmed by western blot (Fig. 4D and S4A). Correspondingly, the ST6GalNAc‐I KO cells showed decreased levels of MUC5AC compared with control (Fig. 4D and S4A). Furthermore, the STn level was also drastically decreased in the ST6GalNAc‐I KO cells compared to control (Fig. S4B). These findings suggest that ST6GalNAc‐I plays a critical role in MUC5AC glycosylation. To address whether STn and MUC5AC protein co‐localize to induce its functional attributes, we performed immunofluorescence (IF) and PLA using STn and MUC5AC antibodies. We observed decreased colocalization of STn and MUC5AC in the ST6GalNAc‐I KO cells compared to A549 control as demonstrated by IF (Fig. 4F) and PLA (Fig. 4G). Overall, the data suggest that ST6GalNAc‐I induces MUC5AC sialylation in LC cells.

### 3.10 Effect of ST6GalNAc‐I on MUC5AC/integrin β4 interaction in lung cancer cells

We have previously reported that MUC5AC interacts with integrin β4 to promote migration of LC cells [25]. Therefore, we wanted to determine the effect of ST6GalNAc‐I on integrin β4 expression as it undergoes O‐glycosylation in cancer [33]. Indeed, ST6GalNAc‐I KO cells drastically reduced the expression of integrin β4 compared with control cells (Fig. 4D and S4A). We also analyzed the expression differences of other glycoproteins, including integrins (α6, β1, β3, and β5) and EGFR family proteins in the KO cells and observed upregulation of integrin β3 alone (Fig. S4C), potentially due to the compensation by other glycosyltransferases. Therefore, we analyzed the expression of other glycosyltransferases in the ST6GalNAc‐I KO cells. Our real‐time PCR results showed that the expression of *GALNT3*, *GALNT5*, and *B3GNT3* was downregulated in the ST6GalNAc‐I KO cells, while *FUT9*, *COLGALT2*, *HAS3*, and *ST8Sia2* were upregulated (Fig. S4D). As the cytoplasmic tail of mucin 1 is demonstrated to negatively regulate the expression of GALNT5 [34], we investigated its expression upon MUC5AC KD A549 cells. We also observed that GALNT5 was negatively regulated by MUC5AC (Fig. S4E,F). Furthermore, ST6GalNAc‐I KO cells showed reduced colocalization of MUC5AC and integrin β4 as compared to control cells as analyzed by immunofluorescence (Fig. 4H) and proximity ligation assay (Fig. 4I). We also observed that colocalization of integrin β4 and STn was completely abrogated in ST6GalNAc‐I KO cells compared to control (Fig. S5A), suggesting that integrin β4 also undergoes sialylation. These results further suggest that the sialylation of MUC5AC and integrin β4 may be essential for their interaction in LC.

### 3.11 Effect of O‐glycosylation inhibitor and a proteasome inhibitor on MUC5AC in ST6GalNAc‐I KO cells

To elucidate the impact of common O‐glycosylation on MUC5AC, we treated ST6GalNAc‐I KO and control LC cells with BAG at various concentrations (0.25 and 0.5 mm) for 72 h. We did not observe any notable difference in the MUC5AC banding pattern with BAG treatments compared to control cells (Fig. S5B). Furthermore, there was no observable difference in the MUC5AC protein levels in ST6GalNAc‐I KO or control LC cells treated with MG132 (5 and 10 mm) for 12 h (Fig. S5C). Overall, these findings suggest that MUC5AC protein expression is not affected by proteosomal degradation in LC cells, and ST6GalNAc‐I plays a critical role in MUC5AC glycosylation.

### 3.12 Effect of ST6GalNAc‐I on the migration of lung cancer cells

**Fig. 5:**
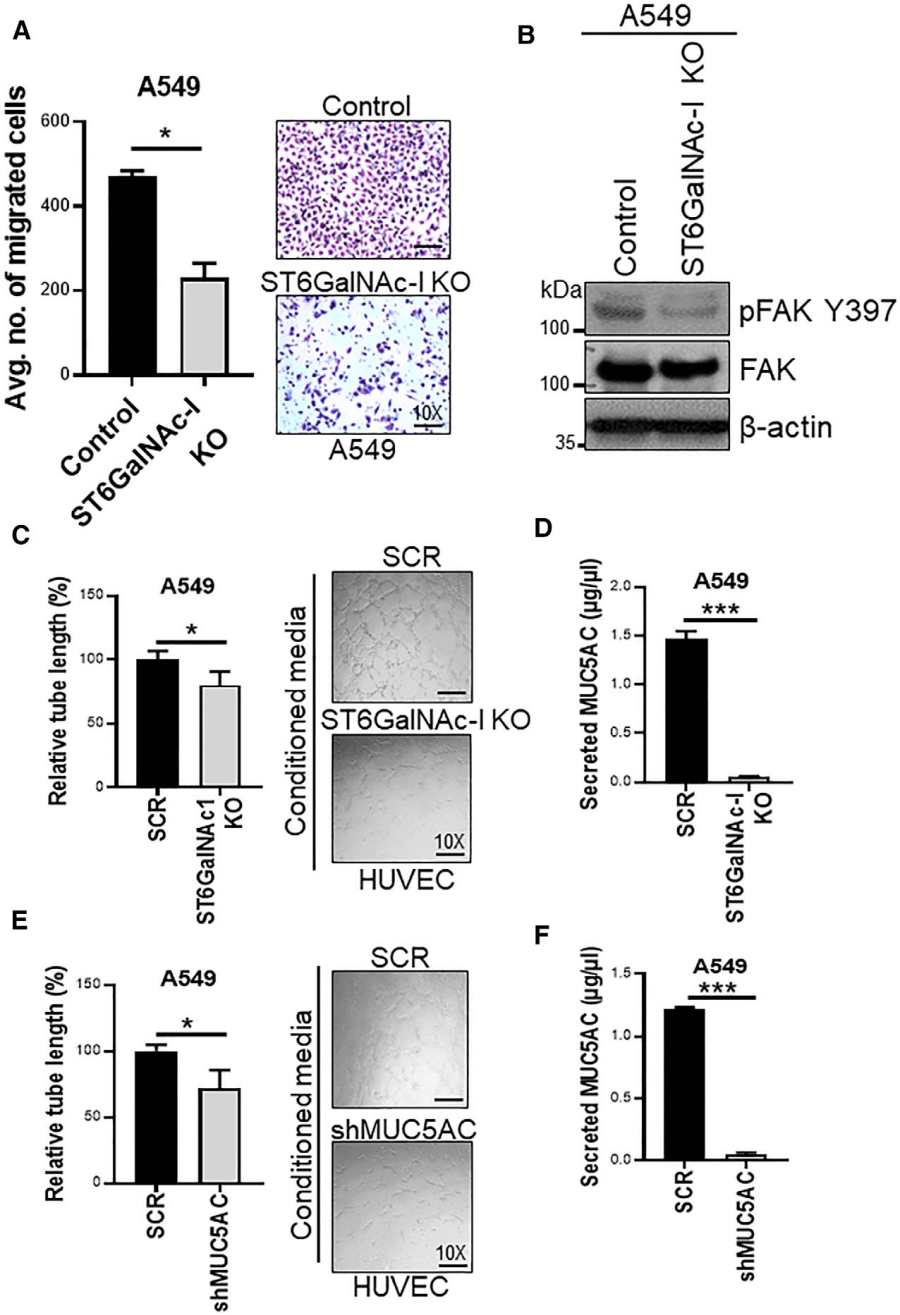
ST6GalNAc‐I is required for lung cancer cell migration and angiogenesis. (A) Bar graph and representative images (right panel) of Boyden chamber transwell migration assay showing decreased migration of ST6GalNAc‐I KO A549 cells compared to control cells (*n* = 3). (B) Western blot analysis showing phosphorylation of FAK (Y397) in ST6GalNAc‐I KO and control cells. β‐actin was used as loading control. (C & E) Bar diagram showing relative tube length of HUVEC cells after treatment with the conditioned media collected from scramble (SCR) and ST6GalNAc‐I KO or shMUC5AC cells (*n* = 3). Representative images of tube formation of HUVEC cells (10×) upon incubation with conditioned media from ST6GalNac‐I KO and MUC5AC KD cells (bottom panel). (D & F) Bar graph showing relative levels of secreted MUC5AC (microgram per microliter) quantified by ELISA in the conditioned media from ST6GalNac‐I KO, MUC5AC KD, and respective control cells (*n* = 3). Statistical significance * *P* < 0.05; *** *P* < 0.001. Error bar represents mean ± SD (*n* = 3). Statistical significance was tested using two‐tailed *t*‐test (A, C‐F).

We then investigated the effect of ST6GalNAc‐I KO in tumor cell migration using the Boyden chamber transwell migration assay. We observed a significant decrease in the migration of ST6GalNAc‐I KO cells compared to control cells (*P* = 0.003) (Fig. 5A). We previously reported that MUC5AC specifically induces phosphorylation of focal adhesion kinase (FAK) at tyrosine 397 in LC cells [23]; however, in the current study, phosphorylation of FAK (Y397) was decreased as a result of ST6GalNAc‐I KO in the LC cells (Fig. 5B and S4A). These findings suggest that the ST6GalNAc‐I/MUC5AC signaling axis may be required for FAK activation during LC cell migration.

### 3.13 MUC5AC is involved in angiogenesis during metastasis

Angiogenesis is an important step in cancer progression and metastasis that involves interactions between cancer and endothelial cells. Study by Bauer *et al*. in the *Muc5ac^−/−^* mouse model clearly demonstrated decreased angiogenesis in lung tumor angiogenesis [35]. Similarly, *in vivo* studies also revealed the angiogenic role of MUC5AC in LC [36]. Therefore, to understand the role of MUC5AC in tube formation, human umbilical vein endothelial cells (HUVEC) were seeded on the Matrigel‐coated plates, and conditioned media collected from ST6GalNAc‐I KO and scramble cells (A549), or shMUC5AC and scramble cells (A549) after 48 h of culture, were added to the cells. The conditioned media from the ST6GalNAc‐I KO (*P* = 0.001) and MUC5AC (*P* = 0.001) knockdown (KD) cells significantly reduced the tube formation of HUVEC cells compared to the respective control cells (Fig. 5C,E). To determine the secretory levels of MUC5AC in the conditioned media, we quantified the supernatants by ELISA and found a significant reduction in the secretory MUC5AC in ST6GalNAc‐I KO (*P* < 0.001) and MUC5AC KD (*P* < 0.001) cells compared to respective control cells (Fig. 5D,F). These results suggest that MUC5AC plays a critical role in angiogenesis during LC pathogenesis.

### 3.14 Role of ST6GalNAc‐I in liver metastasis of LC

**Fig. 6:**
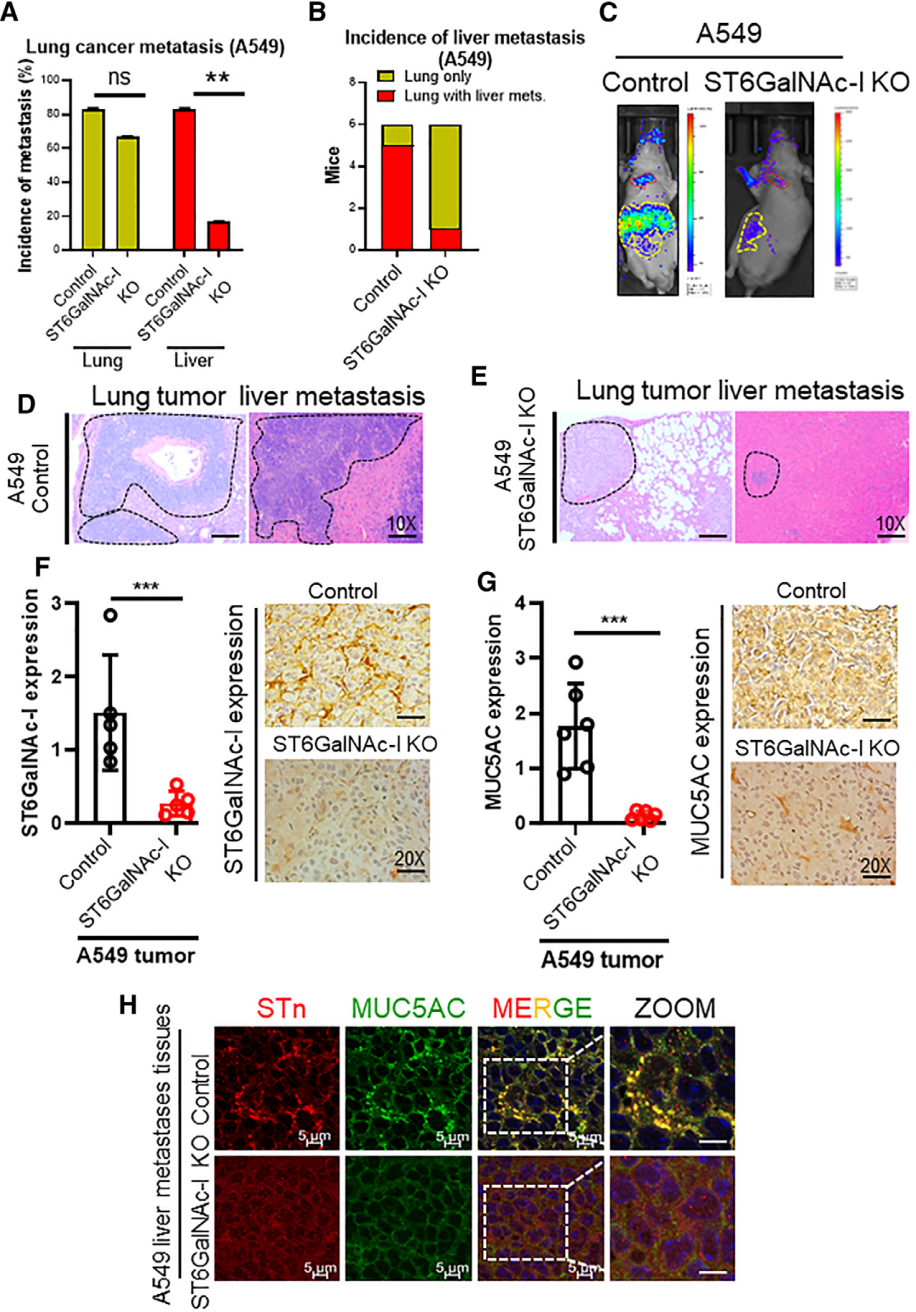
ST6GalNAc‐I mediates lung cancer liver metastasis. (A) Bar diagram representing the incidence of lung and liver metastases after injection of ST6GalNAc‐I KO (4/6) and control (5/6) cells via tail vein in mice during lung tumor development. (B) Bar diagram showing decreased incidence of liver metastasis in mice following injection with ST6GalNAc‐I KO cells (1/6) compared to controls (5/6). (C) Representative IVIS image of control and mice injected with ST6GalNAc‐I KO cells (red mark for lung, yellow mark for liver metastasis). (D & E) Representative histological images showing lung and metastatic liver tumors. (F & G) Bar diagram depicting St6galNAc‐I and MUC5AC expression by IHC in the scramble and ST6GalNAc‐I KO A549 lung tumor xenografts (*n* = 3). Representative images of IHC of ST6GalNAc‐I and MUC5AC expression (10× magnification). (H) Immunofluorescence showing colocalization of MUC5AC and STn in the liver metastatic tissues of control and A549 ST6GalNAc‐I KO xenografts. Statistical significance ** *P* < 0.01; *** *P* < 0.001. Error bars represent mean ± SD of experiments performed in triplicates. Two‐tailed *t*‐test was used to determine the statistical significance (A, F, and G).

Since ST6GalNAc‐I KO showed significant reduction in angiogenesis and migration *in vitro*, we analyzed the role of ST6GalNAc‐I during metastasis. Mice injected with both control (5/6) and ST6GalNAc‐I KO (4/6) cells via tail vein developed tumors in the lungs (*P* = 0.5) (Fig. 6A). However, mice with ST6GalNAc‐I KO cells were less likely to develop liver metastasis (1/5) compared to controls (5/6) (*P* = 0.01) (Fig. 6B). The representative IVIS image (Fig. 6C) and histology of lung tumors, and liver metastasis are shown in Fig. 6D,E, respectively. The metastastic lesions were relatively smaller in mice injected with ST6GalNAc‐I KO cells compared to control. IHC staining of A549 scramble lung xenograft tumor tissue sections exhibited high expression of both ST6GalNAc‐I (Fig. 6F) and MUC5AC (Fig. 6G) compared to ST6GalNAc‐I KO xenografts. We also investigated the angiogenesis markers (VEGR2 and CD31) in the lung tumor xenografts of ST6GalNAc‐I KO and control. We observed decreased expression of angiogenesis markers, VEGFR2 and CD31 in the ST6GalNAc‐I KO compared to control in the lung tumor xenografts indicating the role of ST6GalNAc‐I in angiogenesis (Fig. S5D). These findings suggest that ST6GalNAc‐I/MUC5AC axis is essential for LUAD development.

### 3.15 MUC5AC and its sialylation are required for lung cancer liver metastasis

Since sialylated MUC5AC was decreased in ST6GalNAc‐I KO cells, we wanted to evaluate the expression of MUC5AC and STn in the metastatic liver tissues. Our immunofluorescence study revealed the presence of increased colocalization of MUC5AC and STn in the metastatic tissues injected with control cells compared to tumor tissues with ST6GalNAc‐I KO cells (Fig. 6H), suggesting that MUC5AC and its sialylation may play an important role in the development of liver metastasis.

## 4 Discussion

A better understanding of the biological processes that promote NSCLC metastasis provides better promise for improvement of patient care. Despite recent advances in understanding the pathobiology of LC, mechanisms by which *Kras* and/or *p53* mutations mediate LC progression and metastasis are not well established [7, 37, 38]. Although *Kras* mutations are observed in almost 30% of LUAD [39, 40], targeting this pathway has been a challenge. *p53* tumor suppressor gene is most frequently mutated in cancer and associated with increased invasion and metastatic events [3, 4, 5]. Several GEM model studies have linked *Trp53* mutations with more aggressive and metastatic tumors via targeting various oncogenes [41, 42, 43]

In the present study, we have demonstrated that p53^R175H^ mutation leads to an increased expression of ST6GalNAc‐I resulting in altered glycosylation of MUC5AC that leads to increased LC aggressiveness and the likelihood of liver metastasis. Takamochi *et al*. have demonstrated that ST6GalNAc‐I is significantly overexpressed in LUAD compared to squamous cell LC and plays a critical role in lung carcinogenesis [16]. Previously, we reported that MUC5AC interacts with integrin β4, which is necessary for LC cell migration [25]. Here, we observed that KPA mice, harboring Kras^G12D^ and p53^R175H^ mutations, demonstrate poor overall survival as compared to Kras^G12D^ mutation‐bearing mice (KA). Pathway analysis indicated that enrichment of genes related to cytokines and motility pathways in KPA compared to KA tumor tissues, suggesting that concomitant Kras^G12D^ and p53^R175H^ mutations induce a more aggressive and metastatic phenotype through these pathways. Our transcriptome profile in the KPA and KA autochthonous tumors showed a significant increase of St6galnac‐I in KPA tumors. These findings suggest that p53 mutation mediates tumor aggressiveness, in part via St6galnac‐I. As expected, we also observed higher expression of ST6GalNAc‐I, STn, and MUC5AC in LUAD compared with normal tissues. This is in agreement with previous studies showing that ST6GalNAc‐I is specifically overexpressed in LUAD compared to other subtypes [16], and MUC5AC is increased in LUAD compared to healthy individuals [25].

Glycosylation of MUC plays a critical role in cancer progression [44, 45, 46]. In particular, overexpression of glycosylated MUC contributes to disease initiation, progression, and metastasis [47, 48]. ST6GalNAc‐I is responsible for the synthesis of cancer‐associated antigen STn and correlated with disease progression [15, 49]. STn is found on abnormally glycosylated MUC, which play a major role in the progression of LC [9, 13, 50, 51]. Since ST6GalNAc‐I expression is high in p53^R175H^ mutant LC, we focused to study the mucin profile following mutant p53 transfection. We observed that mutant p53^R175H^‐transfected LC cells expressed higher MUC5AC protein and STn than controls, suggesting that MUC5AC sialylation may require mutant p53^R175H^‐dependent ST6GalNAc‐I. Treatment with O‐glycosylation inhibitor BAG indicated that MUC5AC is O‐glycosylated in LC cells, while treatment with proteasome inhibitor MG132 revealed no effects on the MUC5AC protein stability in mutant p53‐transfected cells.

ST6GalNAc‐I KO resulted in altered glycosylation of MUC5AC in LC cells, suggesting the overall importance of ST6GalNAc‐I in MUC5AC glycosylation. Furthermore, the MUC5AC interaction partner integrin β4 [25] was also decreased in ST6GalNAc‐I KO cells. As MUC5AC and integrin β4 from ST6GalNAc‐I KO cells showed decreased STn content, it is highly possible that its sialylation is critical to mediate MUC5AC and integrin β4 interaction in LC. Furthermore, MUC5AC is known to interact with integrin β4 to mediate LC cell migration via FAK (Y397) phosphorylation [25]. In this study, FAK (Y397) phosphorylation was decreased in ST6GalNAc‐I KO cells, suggesting that ST6GalNAc‐1/MUC5AC axis is involved in FAK signaling. Furthermore, we observed that mice injected with ST6GalNAc‐I KO cells showed less propensity to develop liver metastasis, where MUC5AC level was very low, suggesting that ST6GalNAc‐I and MUC5AC are required for liver metastasis in LC.

## 5 Conclusions

**Fig. 7:**
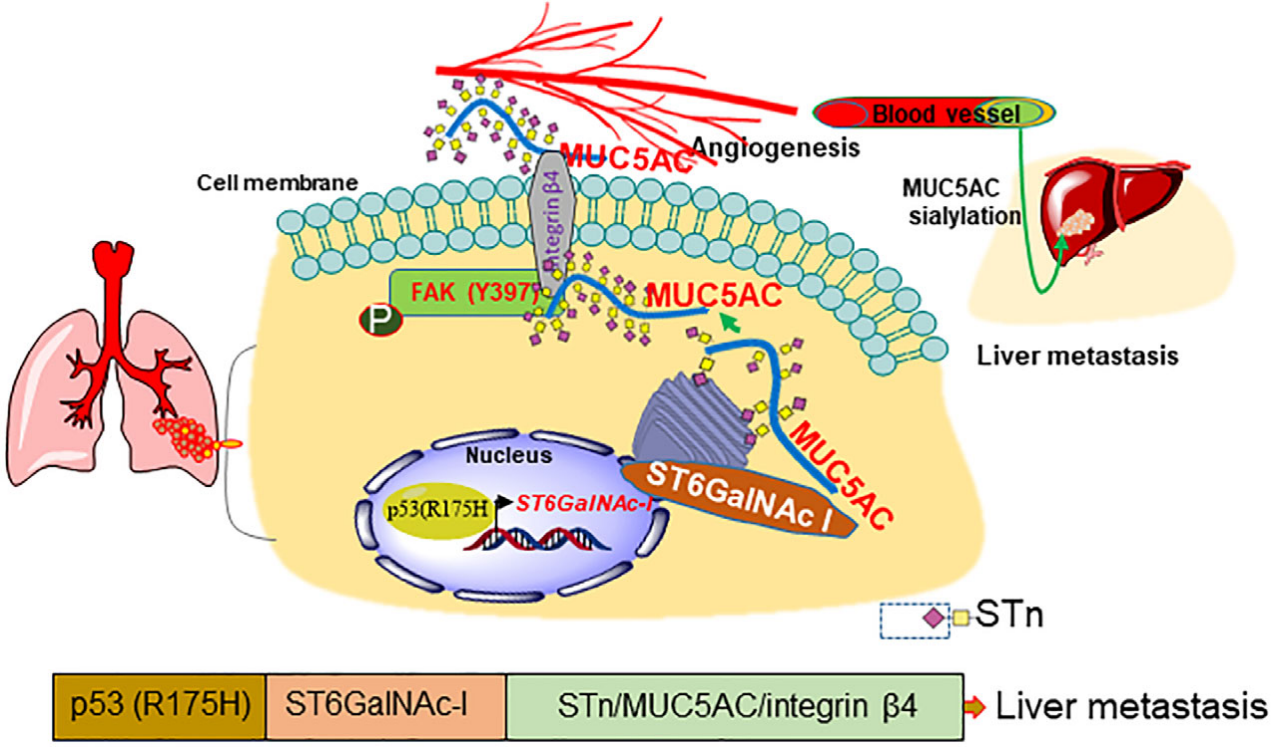
Schema showing the mechanism of ST6GalNAc‐I/MUC5AC axis mediated in lung cancer liver metastasis. This study demonstrated that p53^R175H^ mutation influences ST6GalNAc‐I expression, which leads to MUC5AC sialylation. The sialylated MUC5AC may then promote migration, angiogenesis, and liver metastasis of LC.

Overall, we identified that p53^R175H^ mutation in LC contributes to aggressiveness of the tumor through ST6GalNAc‐I/MUC5AC pathway (Fig. 7). Collectively, our study provides a potential link between mutant p53^R175H^ and ST6GalNAc‐I, which is essential for MUC5AC sialyation in LUAD and promotes aggressive growth and liver metastasis. We posit that MUC5AC and its sialylation play an important role in LC liver metastasis. Our studies in the future will aim to design inhibitors targeting the ST6GalNAc‐I/MUC5AC/integrin β4 axis to prevent LC liver metastasis.

## Conflict of interest

SKB is the co‐founder of Sanguine Diagnostics and Therapeutics, Inc. AKG is a consultant for Flagship Biosciences, AstraZeneca and Genentech; is on the advisory board for AstraZeneca, Genentech, G1 Therapeutics, Blueprint Medicines; and has received research support from Takeda and Oncoceutics. None of the other authors has any conflict of interest.

## Author contributions

Concepts and experiments were designed by IL, SKB, and AKG. Data were predominantly collected and analyzed by IL, SC, PMP, GR, SP, VR, CJ, JR, AP, RC‐V, MS, CS, PN, RS, KM, MWN, KS, LSM, and SML. The manuscript was written by IL with input from SKB, AKG, RS, and PMP and reviewed by all authors. Statistical analysis and IHC scoring were done by SML and LSM, respectively. The sequence of co‐authors is based on their contribution to this project.

### Peer Review

The peer review history for this article is available at https://publons.com/publon/10.1002/1878‐0261.12956.

## Supporting information


**Fig. S1**. Characterization of a spontaneous mouse model of lung tumor.
**Fig. S2**. Mutant p53^R175H^ mediates mucin expression and glycosylation.
**Fig. S3**. Stage‐specific expression of ST6GalNAc‐I and MUC5AC in lung cancer.
**Fig. S4**. Expression of other glycoprotein and glycosyltransferases in ST6GalNAc‐I KO cells.
**Fig. S5**. Colocalization of integrin β4 and STn.

**Table S1**. Primer details.

## Data Availability

The RNA sequence data and materials associated with the current study are available from the corresponding author upon reasonable request.
