# Supplementary material for: ST6GalNAc‐I promotes lung cancer metastasis by altering MUC5AC sialylation
Source: Mol Oncol. 2021 May 1;15(7):1866–81. doi: 10.1002/1878-0261.12956 (PMC8253099; doi:10.1002/1878-0261.12956)
Supplement: Supplementary file 1 — Fig. S1. Characterization of a spontaneous mouse model of lung tumor. Fig. S2. Mutant p53R175H mediates mucin expression and glycosylation. Fig. S3. Stage‐specific expression of ST6GalNAc‐I and MUC5AC in lung cancer. Fig. S4. Expression of other glycoprotein and glycosyltransferases in ST6GalNAc‐I KO cells. Fig. S5. Colocalization of integrin β4 and STn. [file MOL2-15-1866-s002.zip › mol2_12956_Supplementary_Figure_legends_031621.docx]

**Supplementary Figure legends**

**Supplemental Figure 1. Characterization of a spontaneous mouse model of lung tumor.** (**A**) Immunohistochemistry showing Ki67 nuclear positivity in the lung tumors collected from KA and KPA mice (upper panel) (n=3). Representative figures were showing expression of proliferation marker Ki67 in KA and KPA tumor tissues (lower panel). (**B** & **C**) Quantitative PCR data showing significant decrease in squamous cancer marker, *Keratin 5* expression with simultaneously high levels of adenocarcinoma marker, *Keratin 7* in the KA and KPA lung tumors respectively (n=4). (**D**) Clinical significance of *ST6GalNAc-I*, *LY6G6C*, *LTF*, *GDPD3*, *KLK2*, and *CLCNKA* based on expression in TCGA LUAD analyzed in LUNG CANCER EXPLORER (normal (n=59) and LUAD (n=517)). **(E)** Box plot depicting increased expression of *ST6GalNac-I* in TP53 (R175H) mutated LC samples compared to wild-type samples. Statistical significance ** P< 0.01; **** P<0.0001; ns= not significant. Error bars represent the mean ± SD (n=3). Statistical significance were tested using two-tailed *t*-test (**A**) and Ordinary one-way ANOVA followed by Tukey’s multiple comparsion’s test (**B** & **C**).

**Supplemental Figure 2.** **Mutant p53^R175H^ mediates mucin expression and glycosylation.** (**A** & **B**) A549 and H292 cells transfected with mutant p53^R175H^ expressed higher MUC16 compared to control cells (**A**), but MUC4 expression was unchanged (**B**). (**C** & **D**) Immunoprecipitation of MUC4 and MUC16 in mutant p53-transfected cells and probed with STn antibody. (**E-H**) Quantitative PCR showing expression of *NFkB*, *Sp1*, *Gli1,* and *MUC5AC* in the p53 mutant-transfected A549 cells compared to control (n=3). * P< 0.05; ns-not significant. Error bars represent the mean ± SD (n=3). Statistical significance were tested using two-tailed *t*-test (**E-H**).

**Supplemental Figure 3. Stage-specific expression of ST6GalNAc-I and MUC5AC in lung cancer.** (**A**) ST6GalNAc-I expression increased from early stage to later stages in lung adenocarcinoma patients (stage I (81%, 30/37), stage II (88.8%, 16/18), stage III (93.7%, 15/16), and stage IV (100%, 4/4)). On comparison of all stages, the median expression of ST6GalNac-I was highest in stage IV advanced lung adenocarcinoma tissues. (**B**) Stage-specific expression of MUC5AC (stage I (64.8%, 24/37), stage II (88.8%, 16/18), stage III (93.7%15/16), stage IV (100%, 4/4)) in lung adenocarcinoma tissues. (**C**) Heatmap showing co-expression of ST6GalNAc-I and MUC5AC in lung adenocarcinoma tissues.

**Supplemental Figure 4. Expression of other glycoprotein and glycosyltransferases in ST6GalNAc-I KO cells.** (**A**) Western blot analysis showing ST6GalNac-I knockdown in H1437 cells with concurrent decrease in the expression of ST6GalNAc-I, MUC5AC, integrin β4, and phosphorylation of FAK (Y397) compared to control cells. **(B)** STn level was drastically decreased in ST6GalNAc-I KO cells compared to control cells. **(C)** Western blot analysis showing differential expression of integrins in ST6GalNAc-I KO cells compared to controls. HER2 and HER3 levels were decreased in ST6GalNAc-I KO cells compared to controls; no changes were observed for EGFR. β-actin used an internal control. (**D**) Expression of other glycosyltransferases in ST6GalNAc-I KO cells compared to controls (n=3). (**E & F**) Quantitative PCR showing expression of *MUC5AC* and *GALNT5* in A549 scramble and MUC5AC knockdown cells (n=3). Statistical significance * P< 0.05; **** P<0.0001. Error bars represent the mean ± SD (n=3). Statistical significance was tested using two-tailed *t*-test (**E** & **F**).

**Supplemental Figure 5.** **Co-localization of integrin β4 and STn.** (**A**) Immunofluorescence showing co-localization of integrin β4 and STn in the control and ST6GalNAc-I KO LC cells. (**B** & **C**) Western blot analysis showing expression of MUC5AC and integrin β4 after BAG and MG132 treatment in the control and ST6GalNAc-I KO LC cells. β-actin was used an internal control. (**D**) Immunofluorescence showing angiogenesis marker, VEGFR2 and CD31, in the lung xenograft of ST6GalNAc-I KO A549 and control cells.
