# Supplementary figures and images for: ST6GalNAc‐I promotes lung cancer metastasis by altering MUC5AC sialylation
Source: Mol Oncol. 2021 May 1;15(7):1866–81. doi: 10.1002/1878-0261.12956 (PMC8253099; doi:10.1002/1878-0261.12956)

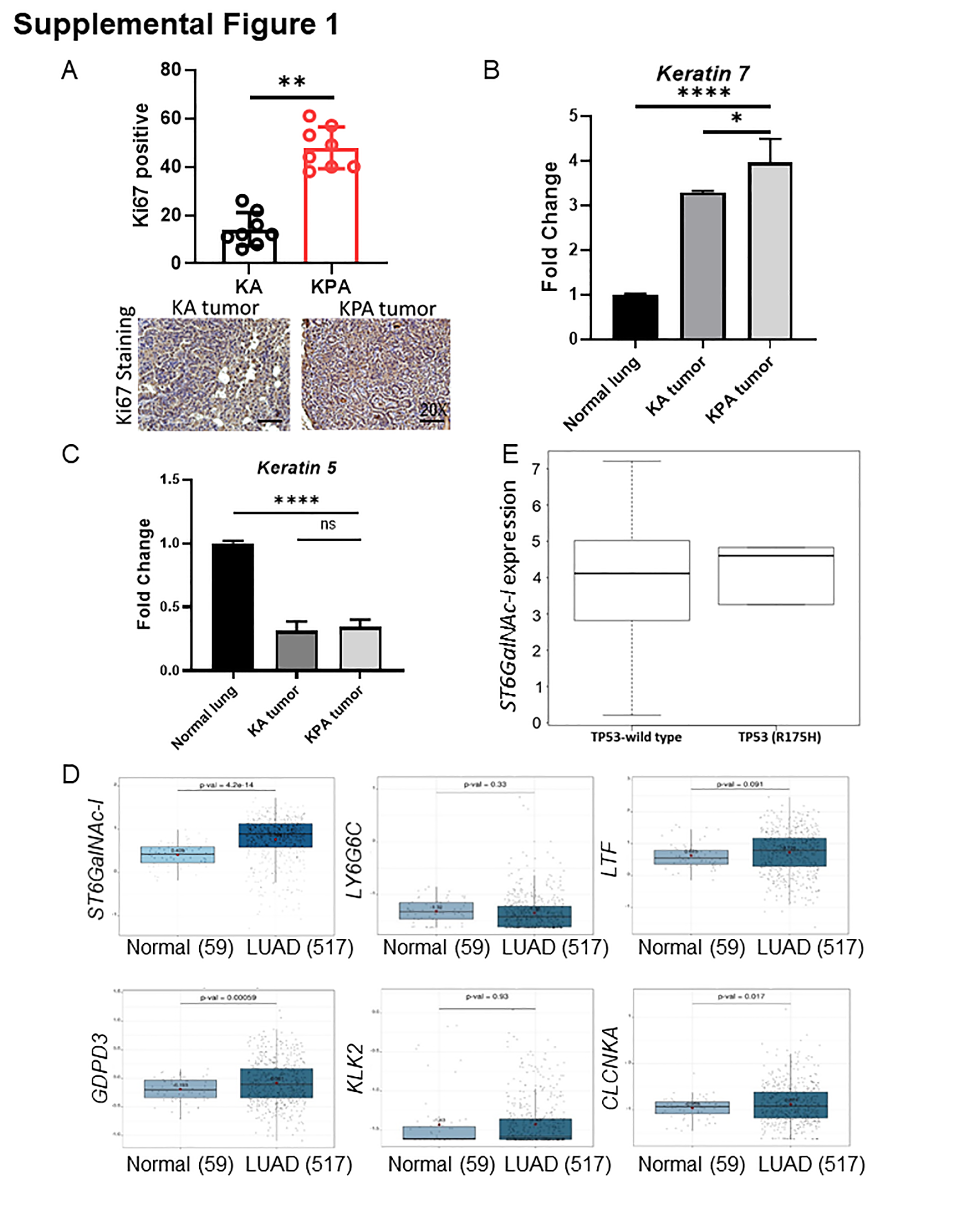

Supplement: Supplementary file 1 — Fig. S1. Characterization of a spontaneous mouse model of lung tumor. Fig. S2. Mutant p53R175H mediates mucin expression and glycosylation. Fig. S3. Stage‐specific expression of ST6GalNAc‐I and MUC5AC in lung cancer. Fig. S4. Expression of other glycoprotein and glycosyltransferases in ST6GalNAc‐I KO cells. Fig. S5. Colocalization of integrin β4 and STn. [file MOL2-15-1866-s002.zip › mol212956-sup-0001-FigS1.TIF]

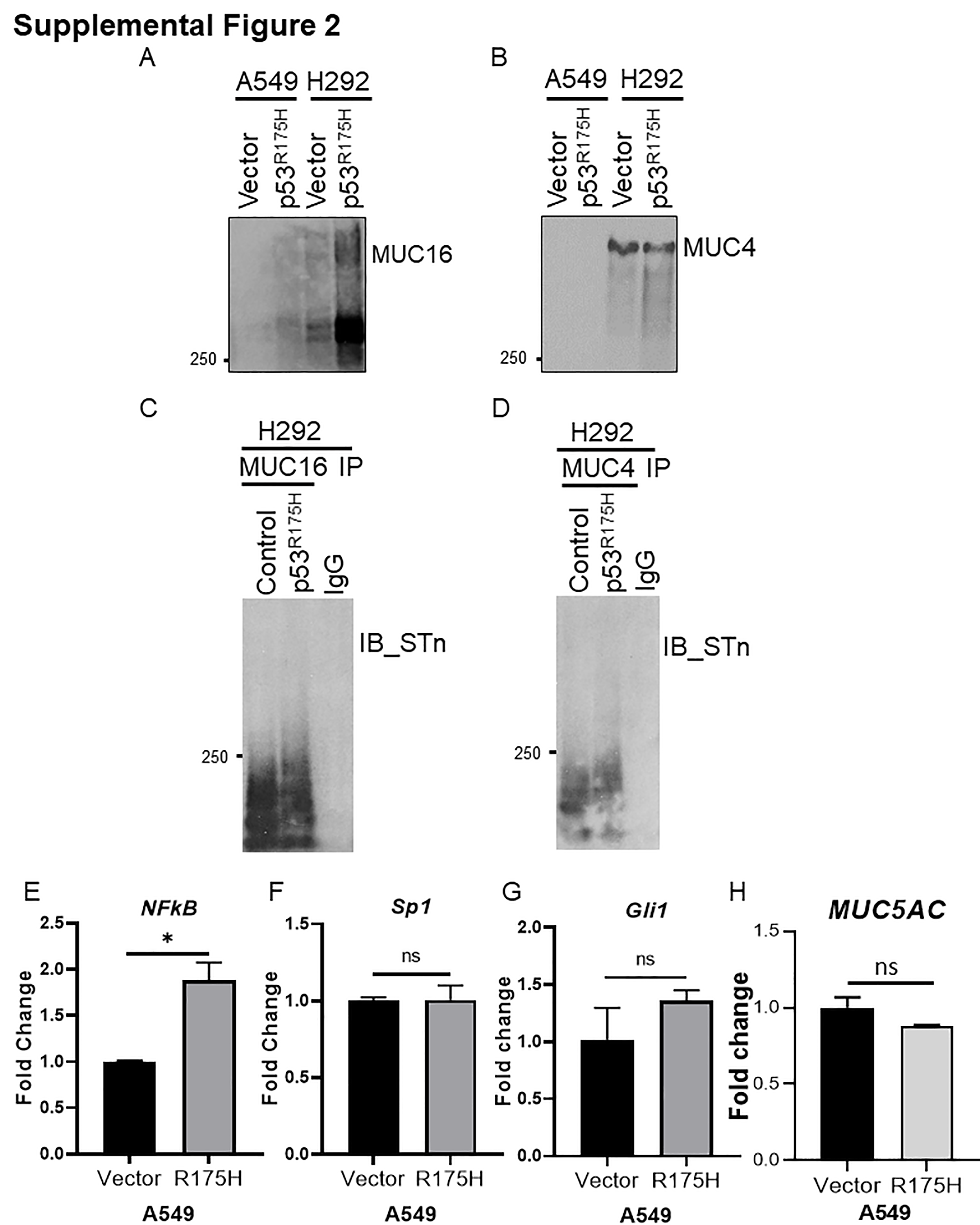

Supplement: Supplementary file 1 — Fig. S1. Characterization of a spontaneous mouse model of lung tumor. Fig. S2. Mutant p53R175H mediates mucin expression and glycosylation. Fig. S3. Stage‐specific expression of ST6GalNAc‐I and MUC5AC in lung cancer. Fig. S4. Expression of other glycoprotein and glycosyltransferases in ST6GalNAc‐I KO cells. Fig. S5. Colocalization of integrin β4 and STn. [file MOL2-15-1866-s002.zip › mol212956-sup-0002-FigS2.TIF]

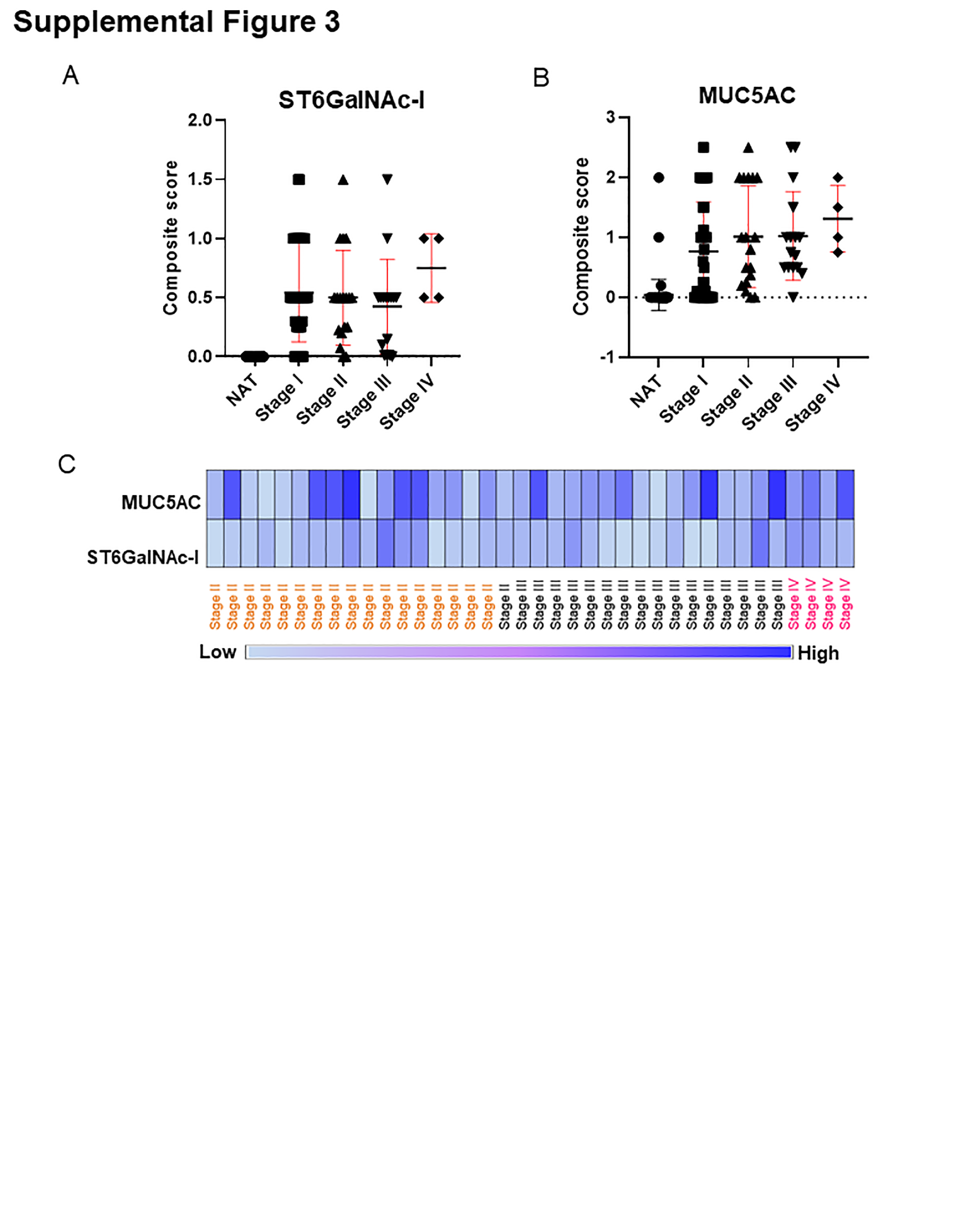

Supplement: Supplementary file 1 — Fig. S1. Characterization of a spontaneous mouse model of lung tumor. Fig. S2. Mutant p53R175H mediates mucin expression and glycosylation. Fig. S3. Stage‐specific expression of ST6GalNAc‐I and MUC5AC in lung cancer. Fig. S4. Expression of other glycoprotein and glycosyltransferases in ST6GalNAc‐I KO cells. Fig. S5. Colocalization of integrin β4 and STn. [file MOL2-15-1866-s002.zip › mol212956-sup-0003-FigS3.TIF]

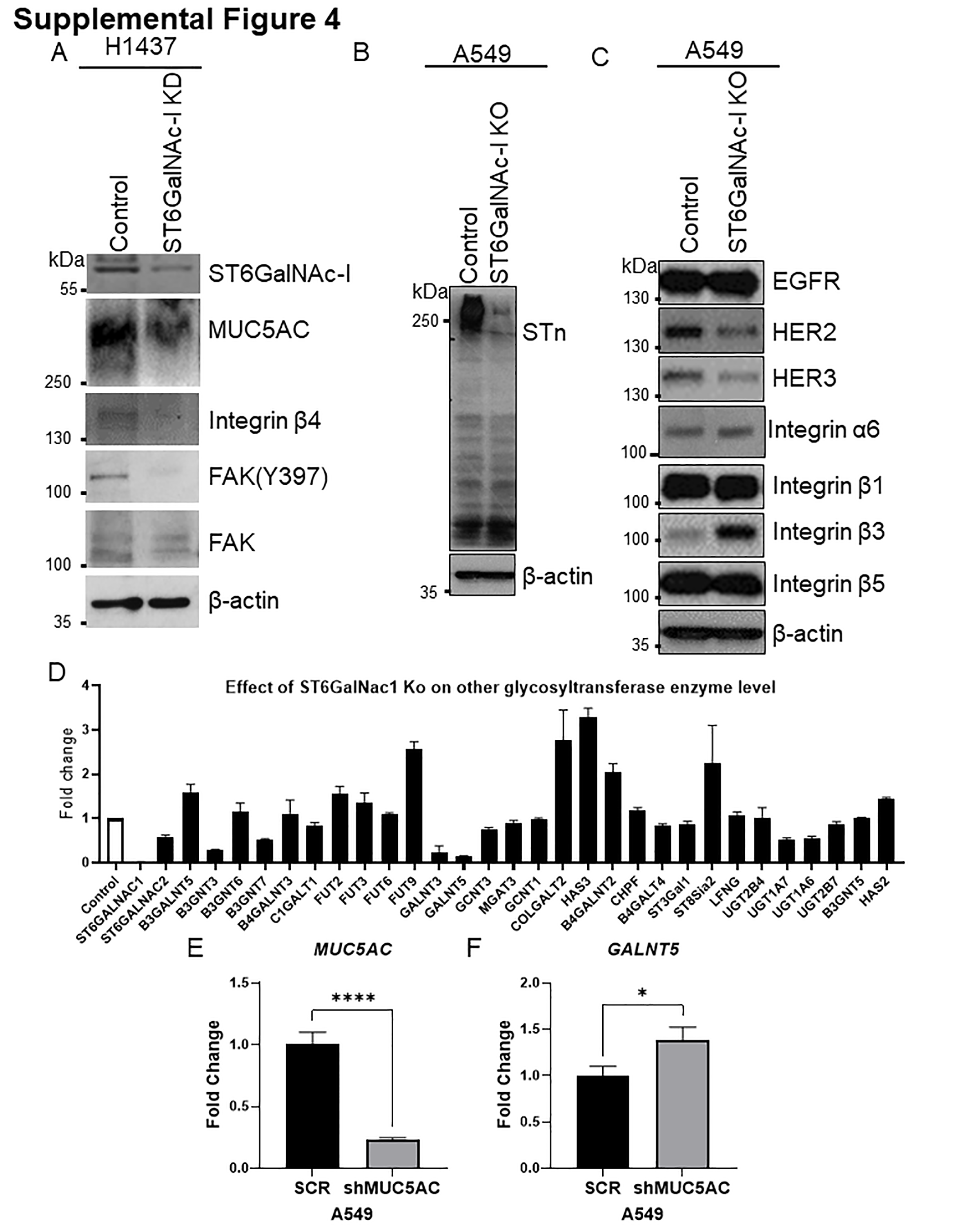

Supplement: Supplementary file 1 — Fig. S1. Characterization of a spontaneous mouse model of lung tumor. Fig. S2. Mutant p53R175H mediates mucin expression and glycosylation. Fig. S3. Stage‐specific expression of ST6GalNAc‐I and MUC5AC in lung cancer. Fig. S4. Expression of other glycoprotein and glycosyltransferases in ST6GalNAc‐I KO cells. Fig. S5. Colocalization of integrin β4 and STn. [file MOL2-15-1866-s002.zip › mol212956-sup-0004-FigS4.TIF]

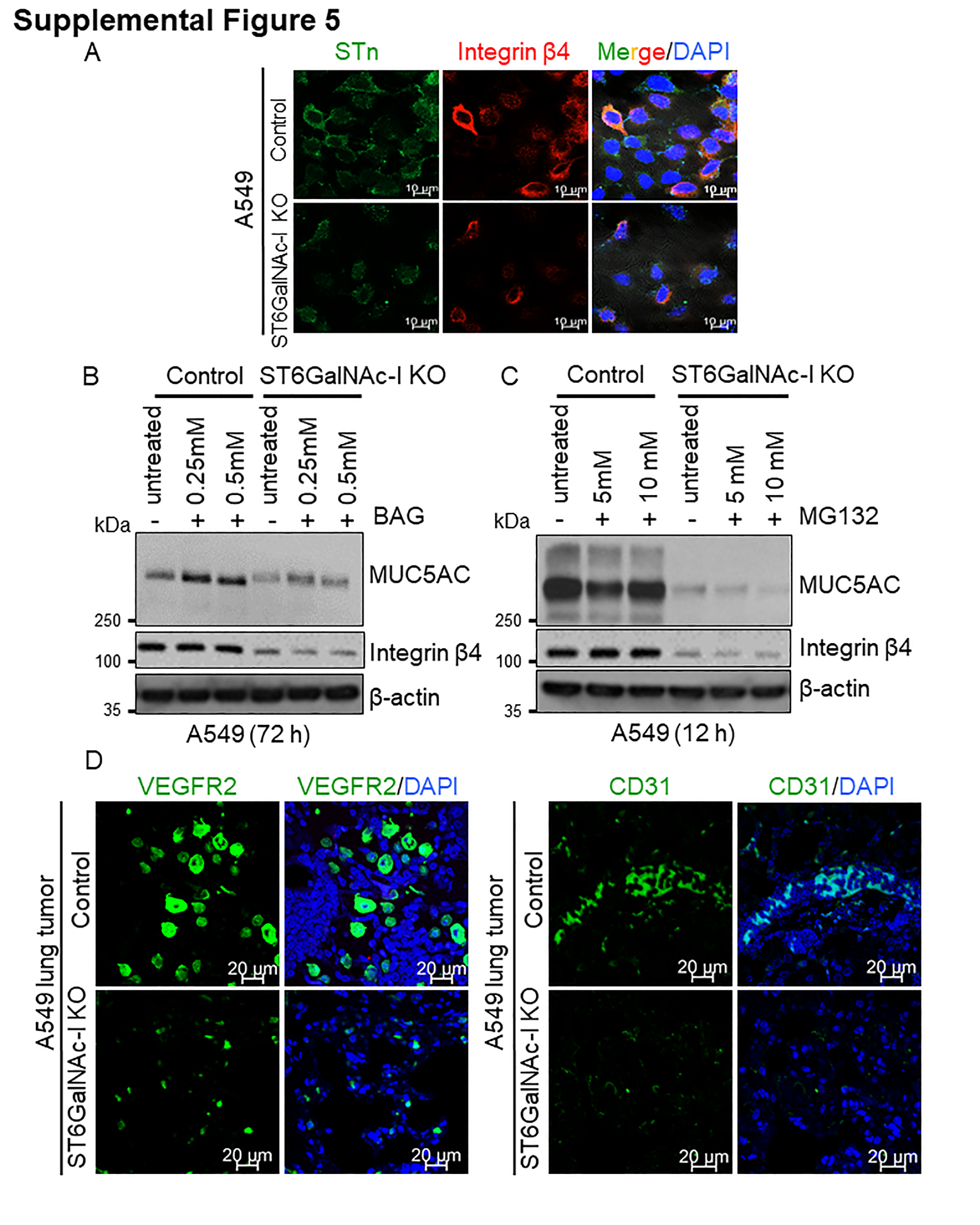

Supplement: Supplementary file 1 — Fig. S1. Characterization of a spontaneous mouse model of lung tumor. Fig. S2. Mutant p53R175H mediates mucin expression and glycosylation. Fig. S3. Stage‐specific expression of ST6GalNAc‐I and MUC5AC in lung cancer. Fig. S4. Expression of other glycoprotein and glycosyltransferases in ST6GalNAc‐I KO cells. Fig. S5. Colocalization of integrin β4 and STn. [file MOL2-15-1866-s002.zip › mol212956-sup-0005-FigS5.TIF]
