# Supplementary material for: ST6GalNAc‐I promotes lung cancer metastasis by altering MUC5AC sialylation
Source: Mol Oncol. 2021 May 1;15(7):1866–81. doi: 10.1002/1878-0261.12956 (PMC8253099; doi:10.1002/1878-0261.12956)
Supplement: Supplementary file 2 — Table S1. Primer details. [file MOL2-15-1866-s001.docx]

| **Sl. No.** | **Primers used** | **Sequence (5'-3')** |
| --- | --- | --- |
| 1 | ST6GALNAC1 RT F | AGGCACAGACCCCAGGAAG |
| 2 | ST6GALNAC1 RT R | TGAAGCCATAAGCACTCACC |
| 3 | ST6GALNAC2 RT F | CTTTGCCCTGTACTTCTCG |
| 4 | ST6GALNAC2 RT R | CAGCACTGGAATGGAGAGA |
| 5 | B3GALNT5 RT F | ACCCTGACTAATACACCTGGA |
| 6 | B3GALNT5 RT R | CCAAACACATCTAGACCACCA |
| 7 | B3GLNT3 RT F | CAAGTACTATGTGCCAGAGGTG |
| 8 | B3GLNT3 RT R | GAAGCGGGACAGCAAGAA |
| 9 | B3GLNT6 RT F | TCAAGAGAACTGAACCACAGG |
| 10 | B3GLNT6 RT R | AGAAGAAATAGACAGGAGCCATC |
| 11 | B3GNT7 RT F | TGTTCCAACGCAGTCTCAC |
| 12 | B3GNT7 RT R | GCCACATCTTTCGGGTTCT |
| 13 | B4GALNT3 RT F | TGTTGAGATGGCACTGAAGAG |
| 14 | B4GALNT3 RT R | TGGAGGTCACAGAGGAAGATG |
| 15 | C1GALT1 RT F | CATCCCTTTGTGCCAGAACACC |
| 16 | C1GALT1 RT R | GCAAGATCAGAGCAGCAACCAG |
| 17 | FUT2 RT F | GATTCAAGCCATGTGGGAGTT |
| 18 | FUT2 RT R | CGGCCTATTGCATTGATCGT |
| 19 | FUT3 RT F | TAGGAGAGGCTGCCATATATCC |
| 20 | FUT3 RT R | CAATTACTGCTTTGCACCCTTG |
| 21 | FUT6 RT F | TGTGGAACCCGCTTTGG |
| 22 | FUT6 RT R | ATTTGGAGACTCAGGTCATGC |
| 23 | FUT9 RT F | CAAGGATTACATCACGGAAAAGC |
| 24 | FUT9 RT R | TGGTCCCAGAACAACAGGTACA |
| 25 | GALNT3 RT F | AAAGCGTTGGTCAGCCTCTA |
| 26 | GALNT3 RT R | AACGAGACCTTGAGCAGCAT |
| 27 | GALNT5 RT F | GAGAAACGGCAGTCTCAATCT |
| 28 | GALNT5 RT R | CACCGAACAGTTCAACATATCAC |
| 29 | GCNT3 RT F | TACTTGTGACCTGCCCTTTAC |
| 30 | GCNT3 RT R | GTTTCCCTTCAGCACCTACA |
| 31 | MGAT3 RT F | GAGTCCAACTTCACGGCTTAT |
| 32 | MGAT3 RT R | GATGTACTCGAAGGTGCCATT |
| 33 | GCNT1 RT F | TGCTTCCTCCACTCGAAACA |
| 34 | GCNT1 RT R | TGTCTTGTGCCCACTCCATC |
| 35 | COLGALT2 RT F | CACAGACCTCAGCTGTACGA |
| 36 | COLGALT2 RT R | GGGCTCGCAGACAGTAGT |
| 37 | HAS3 RT F | CACTGCGGAATTCAAAGCTAAG |
| 38 | HAS3 RT R | GAAAGAGGCGCTGAAGAGAA |
| 39 | B4GALNT2 RT F | CGTGCGGAACGAACTCT |
| 40 | B4GALNT2 RT R | GAGCCGATTCCCACACTC |
| 41 | CHPF RT F | AGGAGCGACCCATTGGA |
| 42 | CHPF RT R | TGTAGGTGGTGTCAGGCA |
| 43 | B4GALT4 RT F | CTGACTTTCCACCTTTCCTACA |
| 44 | B4GALT4 RT R | AATCTCTTGAATGGCACCCA |
| 45 | ST3Gal1 RT F | CAGAGATGGACGGTCACT |
| 46 | ST3Gal1 RT R | CAACTGTGGTTTCTGACG |
| 47 | ST8Sia2 RT F | GAGATCGAAGAAGAAATCGGGAA |
| 48 | ST8Sia2 RT R | GGTGATGAGGAGCCGTTTATTA |
| 49 | LFNG RT F | CCTCTTCCACTCCCACCT |
| 50 | LFNG RT R | CGGCGTTCCGCTTGTTT |
| 51 | UGT2B4 RT F | CTGTGTGGCAACTGTGATATTC |
| 52 | UGT2B4 RT R | CTTCCAGCCTCAGACGTAAT |
| 53 | UGT1A7 RT F | CAGTGAAGACTTACTCAACCTCATAC |
| 54 | UGT1A7 RT R | GCAATGGTGCCGTCCAG |
| 55 | UGT1A6 RT F | CATTCCTAACTGCTCCTCAGAC |
| 56 | UGT1A6 RT R | GCATCAAACTTGCTCTCCTTAAA |
| 57 | UGT2B7 RT F | TTTCACAAGTACAGGAAATCATGTCAAT |
| 58 | UGT2B7 RT R | CAGCAGCTCACTACAGGGAAAAAT |
| 59 | B3GNT5 RT F | GTGGTGCCCCTCCCATTAG |
| 60 | B3GNT5 RT R | GCTCCGGCTGTGTAGTCAGG |
| 61 | HAS2 RT F | CCATTGAACCAGAGACTTGAAAC |
| 62 | HAS2 RT R | TGTGGAAGACTCAGCAGAAC |
| 63 | Sp1 RT F | CCCTTGAGCTTGTCCCTCAG |
| 64 | Sp1 RT R | GTAGCCCCAGAGGAGGAAGA |
| 65 | Gli1 RT F | CTCGGGCACCATCCATTTCT |
| 66 | Gli1 RT R | TGTATTGGCTGCACTCCCTG |
| 67 | NFkB RT F | TGGGAAGGCCTGAACAAATG |
| 68 | NFkB RT R | GTAGTCCACCATGGGATGGG |
| 69 | MUC5AC RT F | GCTCCTCCGAATCCAGCTAC |
| 70 | MUC5AC RT R | CAGTGCTCGGAGAACACGTA |
| 71 | K006 (KRASG12D) | CCTTTACAAGCGCACGCAGACTGTAGA |
| 72 | K005 (KRASG12D) | AGCTAGCCACCATGGCTTGAGTAAGTCTGCA |
| 73 | T035 (Trp53R172H) | CTTGGAGACATAGCCACACTG |
| 74 | T036 (Trp53R172H) | AGCTAGCCACCATGGCTTGAGTAAGTCTGCA |
